# An internet-based mind/body intervention to mitigate distress in women experiencing infertility: A randomized pilot trial

**DOI:** 10.1371/journal.pone.0229379

**Published:** 2020-03-18

**Authors:** Jessica Clifton, Justin Parent, Martin Seehuus, Garyn Worrall, Rex Forehand, Alice Domar

**Affiliations:** 1 Department of Psychological Sciences, The University of Vermont, Burlington, Vermont, United States of America; 2 Department of Psychology, Florida International University, Miami, Florida, United States of America; 3 Department of Psychology, Middlebury College, Middlebury, Vermont, United States of America; 4 Department of Medicine, Larner College of Medicine at The University of Vermont, Burlington, Vermont, United States of America; 5 Boston IVF, Waltham, Massachusetts, United States of America; 6 Department of Obstetrics, Gynecology, and Reproductive Biology, Harvard Medical School, Boston, Massachusetts, United States of America; National Cancer Center Japan, JAPAN

## Abstract

**Objective:**

To determine if an internet-based mind/body program would lead to participants experiencing infertility (1) being willing to be recruited and randomized and (2) accepting and being ready to engage in a fertility-specific intervention. Secondary exploratory goals were to examine reduced distress over the course of the intervention and increased likelihood to conceive.

**Methods:**

This was a pilot randomized controlled feasibility trial with a between-groups, repeated measure design. Seventy-one women self-identified as nulliparous and meeting criteria for infertility. Participants were randomized to the internet-based version of the Mind/Body Program for Fertility or wait-list control group and asked to complete pre-, mid- and post-assessments. Primary outcomes include retention rates, number of modules completed, and satisfaction with intervention. Secondary exploratory outcomes sought to provide preliminary data on the impact of the program on distress (anxiety and depression) and self-reported pregnancy rates relative to a quasi-control group.

**Results:**

The retention, adherence, and satisfaction rates were comparable to those reported in other internet-based RCTs. Although time between pre- and post-assessment differed between groups, using intent-to-treat analyses, women in the intervention group (relative to the wait-list group) had significant reduction in distress (anxiety, p = .003; depression, p = .007; stress, p = .041 fertility-social, p = .018; fertility-sexual, p = .006), estimated as medium-to-large effect sizes (ds = 0.45 to 0.86). The odds of becoming pregnant was 4.47 times higher for the intervention group participants as compared to the wait-list group, OR 95% CI [1.56, 12.85], p = .005 and occurred earlier. The findings suggest that the research design and program specific to this population are feasible and acceptable. Replication efforts with an active control group are needed to verify distress reduction and conception promotion findings.

## Introduction

Infertility is a frequently occurring chronic health condition which can persist throughout the reproductive years [1]. As is characteristic of most chronic health conditions, heightened emotional distress (i.e., anxiety and depression) is often comorbid with infertility diagnoses [2]. The uncertainty of infertility and reproductive medical treatment can evoke (or perpetuate) anxiety, whereas the sense of loss and loss of control over the desired outcome can lead to or exacerbate feelings of depression and hopelessness [3].

Women who experience comorbid infertility and emotional distress are a particularly high-risk group. Distress (i.e., anxiety and depression) is present for 25–53% of women prior to starting and 40–75% during reproductive medical treatment [4,5]. Emotional distress can lead to couples giving up on their dreams to conceive often by choosing not to seek out additional medical support and/or discontinuing reproductive medical services [6,7]. Women who persist on their reproductive journey are at risk for increased symptom severity [8]. Furthermore, their symptoms may outlast the infertility diagnosis continuing through pregnancy resulting in potential long-term negative effects on the child and mother [3,8–10].

Previous research has found that 9–21% of women receiving medical care for infertility reported currently seeing a therapist or other mental health professional [5,11,12]. Based on these data, the rate of women experiencing emotional distress due to infertility far exceeds the rate of women getting this needed professional support. Barriers that may account for this discrepancy include stigma, such as fear of being dismissed from reproductive treatment, skepticism regarding efficacy, cost of care, uncertainty of who to contact, challenges regarding travel, and difficulty scheduling [11,13,14].

Although women experiencing infertility need and are often open to additional support, the barriers to face-to-face psychotherapy are many and strong. Consequently, women diagnosed with infertility need professional support that is private, convenient, and effective. Internet-based interventions are a promising modality of care that can address these barriers and have shown to be effective in reducing emotional distress [15]. Research has shown that this method can be especially appealing for individuals experiencing infertility [16,17].

The goal of this Stage I pilot study was to translate an empirically tested face-to-face third-wave cognitive-behavioral therapy [18] specific to infertility, The Mind/Body Program for Fertility [19–22], into an internet-based intervention to suit the needs of this population. Similar to the goals of other internet-based intervention pilot studies, we describe the translation process of converting a face-to-face intervention into an internet-based program. Additionally, we sought to demonstrate that (a) an acceptable number of women experiencing infertility could be identified, recruited, and would be willing to be randomized to the intervention or wait-list groups and (b) participants would report appropriate levels of acceptance and readiness to engage in the intervention as measured by retention rates, treatment adherence, and treatment satisfaction. As secondary goals, we examined whether participants (relative to a quasi-wait-list control group) would demonstrate reduced distress over the course of the intervention (i.e., anxiety, depression, perceived-stress, infertility-related distress) and whether pregnancy rates in the intervention group would be higher. Our wait-list group is a quasi-control as the length of time between the pre- and post- assessments of the two groups was not matched, although the wait-list control was matched by demographics.

## Materials and methods

### Participants & procedures

The study was a randomized controlled trial, using a between groups repeated measures experimental design, conducted between March 2015 and April 2017. The sample size was set at a minimum of thirty participants based on feasibility and recommendations for pilot studies preceding clinical trials [23]. To account for attrition, we sought to recruit twice the recommendation. Inclusion criteria included the following: (a) at least 18 years old, (b) nulliparous, (c) Internet access, (d) English-speaking; (e) no psychotropic medication changes in the last month; (f) had not completed nor was currently engaged in a Mind/Body Program for Fertility; and (g) no suicidal ideation/intent, psychotic disorder, eating disorder, substance abuse or dependence [assessed by an internet-based MINI International Neuropsychiatric Interview for DSM-IV, 24, 25]. Participants were recruited through flyers posted in reproductive care offices throughout the United States, social media postings, Amazon Mechanical Turk^®^, and postings on infertility-related websites (i.e., Resolve.org). Ninety-five percent of participants reported living in the United States.

Qualified and interested participants who passed an initial eligibility criteria screener and completed the baseline questionnaires pre-intervention were randomized to either the internet-based intervention or a quasi-control wait-list condition. Randomization was determined by a random number generator via Random.org [26]. An electronic file with the randomization sequence was created prior to recruitment and maintained by co-author Garyn Worrall, M.D. Both groups completed a mid- and post-assessment. The mid-assessment was emailed to participants at 5-weeks and post-assessment at either 10-weeks (control group) or at the end of the mind/body program (intervention group). In addition to the mid- and post-assessments, participants were asked to email the researchers when or if they became pregnant and were asked about pregnancy status at mid-, post- and 1-year follow-up. Wait-list participants received $10 for completing the online pre-assessment and $15 upon completion of the post-assessment as an incentive to complete the study. As further incentive, wait-list participants were offered the opportunity to engage in the internet-based intervention after completing the post-assessment. This work was funded by the Department of Psychological Science at the University of Vermont (UVM) and approved by the UVM Committees on Human Research (CHRMS #14-293) and all participants provided consent. Since this study was originally conceived and conducted as a pilot, it was registered retrospectively (Clinicaltrails.gov: NCT03343405), as described by the NIH (https://prsinfo.clinicaltrials.gov/ElaborationsOnDefinitions.pdf).

### Measures

#### Retention, adherence, and satisfaction

Participants who completed the mid-assessment were considered retained. Comparable to other internet-based studies, participants in the intervention group who completed five or more (out of ten) modules were considered to have adhered to the intervention [16,17]. At the beginning of every module participants were asked if they completed the practice exercises since the last module. Additionally, at the end of each of the ten intervention modules, participants were asked to rate their ability to complete each module and the module’s helpfulness. At post-assessment, participants were given the Client Satisfaction Inventory Short-Form [CSI-SF; 27], a 9-item self-report scale that assesses the satisfaction of the participant with the effects of treatment. Participants are asked to rate the way they feel about the services they received on a 7-point Likert scale, ranging from 1 (“none of the time”) to 7 (“all of the time”). The scoring range is between 0 and 100, with higher scores indicating more satisfaction [for scorign conversion see, 27]. Additionally, participants were asked at post-assessment how helpful each element (i.e., therapist feedback, relaxation exercises, progress log) of the intervention were as well as open-ended questions regarding the advantages and disadvantages of the intervention.

#### Psychological distress

Participants completed online self-report assessments at three time-points (screener/pre-, mid-, and post-assessment). These assessments included two primary measures: The Beck Anxiety Inventory [BAI; 28, 29], a 21-item measure used to assess somatic or panic-related anxiety symptoms, and the Beck Depression Inventory-II [BDI; 30, 31], a 21-item measure used to assess cognitive, affective, and physical depressive symptoms. Additionally, participants completed the Perceived Stress Scale [PSS; 32], a 10-item questionnaire measuring the present level of self-rated stress in the last month, and the Fertility Problem Inventory [FPI; 33], a 46-item questionnaire that measures domains considered important in understanding specifically perceived infertility related stress. The FPI has a global index and five subscales which include social concern, sexual concern, relationship concern, need for parenthood, and rejection of childfree lifestyle. In the current sample, Cronbach alpha reliability coefficients for all measures ranged from 0.88 to 0.98.

#### Pregnancy rates

Pregnancy rates were reported throughout the course of the study period (i.e., direct emails, during the modules, during the expected assessment periods).

### Summary

In summary, (a) willingness was determined by the number of women randomized; (b) acceptance and readiness were determined by the number of participants that completed the mid- and post- assessments, completed five or more modules (intervention group only), and satisfaction ratings collected after each module and at post-assessment; (c) distress measures were collected at pre-, mid-, and post-assessment; and (d) pregnancy rates were reported throughout the course of the study period.

### Intervention

The internet-based intervention was designed to mirror the face-to-face Mind/Body for Fertility program in structure and content [20,34]; thus, as with the 10-week face-to-face protocol, this internet-based intervention included information and exercises structured into ten separate modules each of which was designed to take less than an hour to complete. Each module included text, audio, video, interactive elements, and electronic downloads for homework completion [for more information, see 34]. Both assessments and modules were developed using an open-sourced online survey tool, LimeSurvey 2.0 [35]. As recommended, therapist feedback was provided after the completion of the screener/pre-assessment, after each module, and the therapist was available by email to answer any questions or concerns [36].

Participants assigned to the intervention condition were asked to sequentially complete the self-guided 10-module program and assigned practice exercises. The skills and strategies taught included: (a) knowledge regarding the relationship between stress, lifestyle, and fertility; (b) relaxation techniques including diaphragmatic breathing and Hatha Yoga; (c) mindfulness; (d) cognitive restructuring; (e) stress reduction strategies; (f) listening and communication skills; (g) strategies for emotional expression and effective coping with anger; and (h) assertiveness training and goal-setting skills. Similar to the face-to-face program, homework was assigned at the end of each module and included: tracking of health-related information (i.e., alcohol use), time participating in relaxation exercises (2x a day for 20 minutes), and CBT tools (i.e., cognitive restructuring). For intervention participants that completed all ten modules, an estimated six total clinician hours were spent reviewing participant modules responses and writing feedback delivered to participant via email [34].

### Data analysis

The intervention group and quasi-control wait-list group were compared on demographic and primary study outcome variables collected at baseline to identify randomization failures (see Table 1). No such differences were identified. To assess group differences in within-person symptom changes over time we utilized latent change score (LCS) models using Mplus [37,38]. Both between- and within-group effect sizes (ESs) were calculated using Cohen’s d [39]. To account for non-normality and missing data, maximum likelihood estimation with robust standard errors was deployed in order to include all randomized participants in the proposed analyses. All participants who were randomized were included in the analyses using full information maximum likelihood estimation. Binary logistic regression using SPSS [40] was used to determine odds ratios of group pregnancy rates. Additionally, an exploratory analysis using a non-parametric model of Kaplan-Meier was used to estimate the rates of pregnancy while accounting for participants lost to follow-up or censored (i.e., determined not pregnant) at study close. “Survival” was defined as the duration in the study until becoming pregnant. The time unit was the number of days from pre-assessment to pregnancy.

**Table 1:** Baseline demographic characteristics.

Characteristics	Intervention (n = 36)	Wait-list (n = 35)	*t*/χ	p-value
**Basic Demographics**				
Age (y), mean (SD)	32.83 (3.56)	33.49 (3.97)	0.73	0.47
Single/separated	5.70	2.90	0.32	0.57
Relationship duration (y), mean (SD)	8.62 (4.07)	8.03 (3.83)	-0.61	0.54
Heterosexual	94.10	94.10	0.38	0.54
College educated	97.30	94.30	0.00	0.98
Living in a rural area	19.40	25.70	0.40	0.53
Income < $90k, %	49.90	63.70	0.03	0.87
Ethnicity, White %	83.30	77.10	0.43	0.51
Full-time employment %	86.10	80.00	0.14	0.71
**Fertility**				
Months trying, mean (SD)	34.47 (26.52)	28.53 (13.28)	-1.29	0.20
Treatment covered by insurance, %	63.90	54.30	0.68	0.41
Previous miscarriages	29.00	22.00	0.23	0.63
Infertility Diagnosis			0.69	0.88
Male-factor	8.30	11.40		
Female-factor	36.10	28.60		
Combined (Female/Male Factor)	19.40	20.00		
Unexplained	33.30	40.00		
Unknown	2.80	0.00		
Baseline Stage of Treatment, %			7.46	0.19
None	5.60	2.90		
Diagnostic Testing	8.30	22.90		
Medication/Injections	2.80	14.30		
Intrauterine Insemination	27.80	14.30		
Waiting to start IVF	25.00	22.90		
Started IVF	30.60	22.90		

None of the comparison tests were statistically significant (continuous data = t-test; categorical data = chi-square). SD = Standard deviation. IVF = In vitro fertilization.

## Results

### Recruitment and randomization

**Fig 1:**
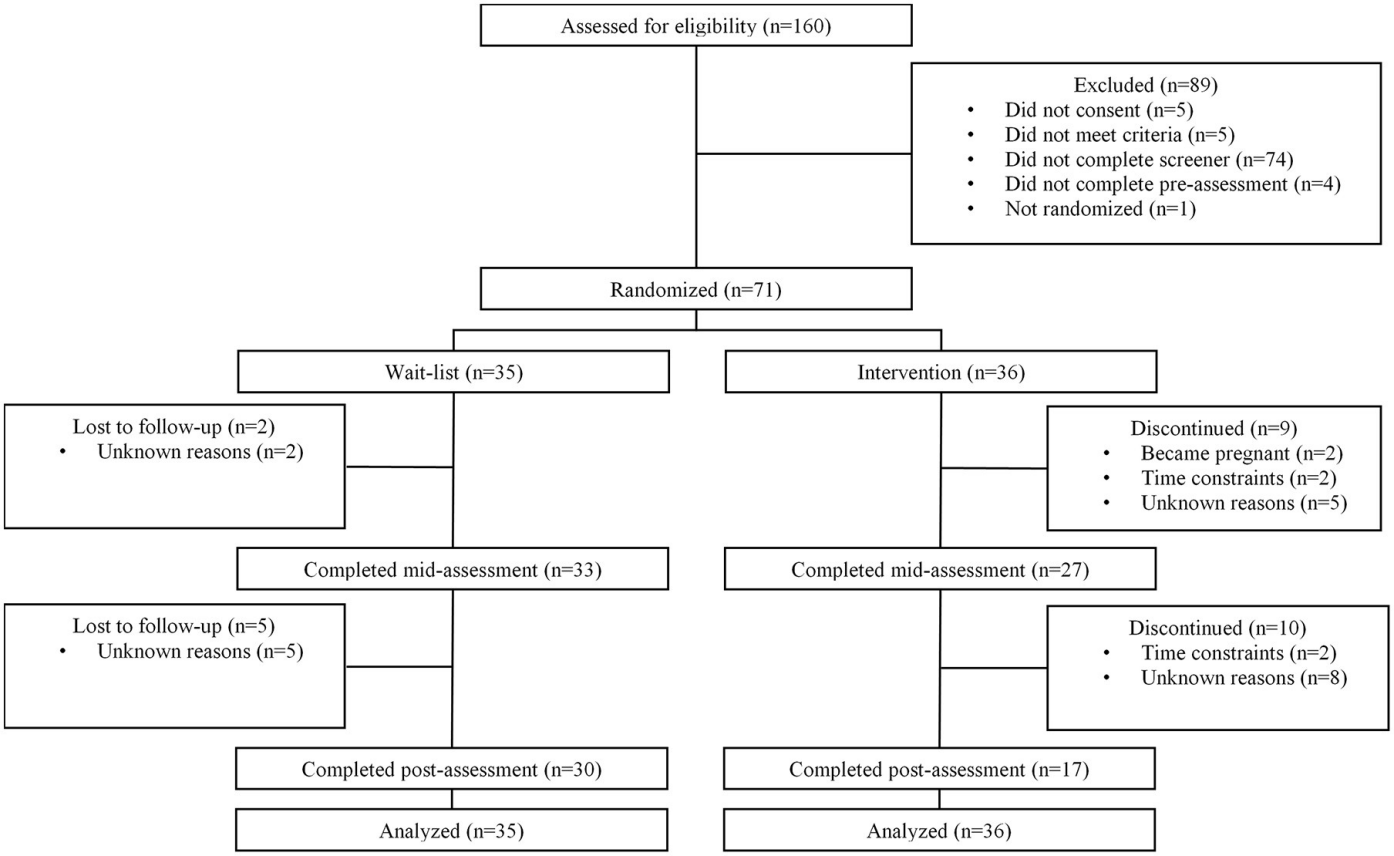
CONSORT diagram.

A total of 71 participants were randomized (see Fig 1 for complete details). Demographic and medical characteristics appear in Table 1. Eighty-four percent of participants did not meet current criteria for a psychiatric diagnosis, 5.6% met criteria for Major Depressive Disorder, 7.0% for an anxiety disorder, and 1.4% meeting criteria for both. Approximately 17% of participants reported taking psychiatric medication(s), including SSRI’s (*i*.*e*., Sertraline, Fluoxetine), NDRI’s (*i*.*e*., Bupropion), benzodiazepines, and medication for ADHD. Twenty-six (38%) participants reported seeing a counselor or therapist. Of these participants, 65% reported seeing a therapist to help them cope with reproductive-distress. Although no participants had completed a formal Mind/Body for Fertility program, 39% of participants reported engaging in at least 70 minutes of some type of relaxation exercise on average (i.e., yoga, breath-focus, mindfulness, prayer, tai-chi, etc.) per week (estimated 10 minutes a day) with many participants reporting yoga.

### Acceptance to engage in internet-based intervention and pilot study

#### Retention and adherence

Mid-assessment and post-assessment completion rates can be seen in Fig 1. The time between pre- and post-assessment significantly differed between the intervention (M = 233.97, SD = 143.5) and wait-list (M = 90.73, SD = 32.71) groups, F (1, 44) = 28.48, p < .001.

Almost all (97%) participants in the treatment group completed module one, 69% module two, 67% module three and four, 61% module five, 58% module six, 42% modules seven through nine, and 39% (n = 14) module ten. On average, participants completed four modules. Participants were encouraged but not required to complete one module per week; participants waited on average 21 days (SD = 16.36) to complete the first module. This pattern continued throughout the program, with the average total duration of the intervention of 6 months (*M* = 184 days, SD = 35.86 days). Participants reporting higher levels of general stress at baseline were more likely to drop-out after the first module, OR = 1.47, 95% CI [1.10, 1.96], *p* = .009, but not later modules (all *p*s > .05). All wait-list participants who completed the post-assessment expressed interest in the intervention, although only a minority (17%) got as far as module ten.

Amongst intervention participants who indicated they did not want to participate any longer, two reported it was due to being pregnant and four because of time constraints. Nineteen participants in the study did not complete the mid- and/or post-assessment without specifying a reason and were lost to follow-up (see Fig 1).

#### Satisfaction

Most participants in the intervention group reported that the modules were moderately challenging and helpful. Ninety-eight percent of participants reported that the modules were at least slightly helpful and at least 60% reported that the modules were moderately to very helpful. Eighty-two percent (SD = 12.64%) of treatment completers were satisfied with the intervention overall (as measured by the CSI).

On average, participants reported that the Mini and other relaxation exercises, therapist feedback, and partner communication exercises were the most helpful (mean score 8 out of 10); whereas daily thoughts/emotions logs and tracking of homework activities were reported to be least helpful (mean score 6 out of 10). Advantages of the program that emerged from free-text participant responses included finding the content/resources/therapeutic feedback “validating” and “helpful”; the relaxation techniques “effective”; and praising the programs “flexibility” and “convenience.” Some disadvantages/changes offered surrounded the operational structure of the modules, including timing, duration, order, and release of modules. Specifically, a few participants suggested that there should be “fewer” modules; “shorter” modules; modules should be “released at consistent days/times” (versus after completion of a previous module); an option to pick and choose the modules (versus having to complete them in a certain order); and higher quality videos/audio. Overall the quantitative and qualitative data suggest that participants were satisfied with the program.

#### Secondary outcomes

The pre- and post-assessment means and within-group ESs (Cohen’s *d*) for the intervention and quasi-control wait-list groups as well as the between-group ESs are shown in Table 2. Sample statistics reported in Table 2 and 3 were calculated using ML with Mplus 7.3 [37] following intent-to-treat guidelines [41,42]. Within-group ESs for the intervention group were medium-to-large for change in depressive symptoms, anxiety symptoms, infertility related stress specific to sexual and social concerns, and overall perceived stress (*d*s range from .49 to .86) whereas the quasi-control wait-list group averaged near-zero change across outcomes.

**Table 2:** Within-group comparisons.

	Intervention			Control		
	Pre	Post		Pre	Post	
	Mean (SD)	Mean (SD)	*d*	Mean (SD)	Mean (SD)	*d*
BDI	14.86 (9.29)	9.27 (6.27)	0.72	16.68 (9.79)	15.73 (12.29)	0.09
BAI	9.89 (7.57)	6.34 (6.78)	0.50	10.94 (7.39)	11.33 (7.73)	-0.05
PSS	20.33 (6.92)	16.90 (5.02)	0.57	21.74 (7.02)	21.24 (8.47)	0.07
FPI Total	165.94 (26.10)	167.35 (72.76)	-0.03	170.66 (30.04)	163.44 (27.34)	0.25
Social	41.14 (75.45)	35.67 (13.79)	0.49	41.45 (8.98)	40.73 (8.75)	0.08
Sexual	25.84 (7.70)	17.74 (11.07)	0.86	27.63 (7.77)	28.41 (8.03)	-0.10
Relationship	27.07 (7.77)	37.49 (43.74)	-0.40	27.94 (7.38)	28.58 (6.89)	-0.09
Childfree	30.22 (9.33)	29.89 (20.81)	0.02	31.19 (74.61)	29.77 (8.83)	0.16
Parenthood	42.43 (7.57)	40.42 (11.37)	0.21	42.46 (9.93)	41.48 (9.93)	0.10

BDI = Beck's Depression Inventory-II; BAI = Beck's Anxiety Inventory; PSS = Perceived Stress Scale; FPI = Fertility Problem Inventory (Five Subscales: Social Concern, Sexual Concern, Relationship Concerns; Rejection of Childfree Lifestyle; Need for Parenthood). *d* = Cohen's d effect sizes.

**Table 3:** Between-group comparisons.

	B	b lower	b upper	d	d upper	d lower	p value
BDI	-7.98	-13.77	-2.20	-0.86	-1.48	-0.24	0.01
BAI	-4.86	-8.02	-1.70	-0.67	-1.11	-0.23	0.00
PSS	-4.15	-8.77	0.47	-0.61	-1.30	0.07	0.08
FPI Total	-6.14	-29.19	16.91	-0.12	-0.58	0.34	0.60
Social	-5.12	-9.34	-0.89	-0.45	-0.83	-0.08	0.02
Sexual	-6.13	-10.51	-1.74	-0.64	-1.10	-0.18	0.01
Relationship	4.71	-10.43	19.85	0.19	-0.41	0.78	0.54
Child	-2.91	-8.13	2.32	-0.20	-0.55	0.16	0.28
Parenthood	-1.55	-4.74	1.64	-0.15	-0.45	0.15	0.34

BDI = Beck's Depression Inventory-II; BAI = Beck's Anxiety Inventory; PSS = Perceived Stress Scale; FPI = Fertility Problem Inventory (Five Subscales: Social Concern, Sexual Concern, Relationship Concerns; Rejection of Childfree Lifestyle; Need for Parenthood). B = regression coefficient. b = mean difference. d = Cohen's d effect size.

LCS models supported findings from the above within-group ESs with participants randomized to intervention evidencing significant decreases in anxiety, depression, and infertility related social and sexual stress outcomes compared to the quasi-control wait-list group (see Table 3). Additionally, greater reductions in overall perceived stress were observed for the intervention as compared to wait-list control group (though the unstandardized beta coefficient was marginally significant, *p* = .078, the standardized beta coefficient was statistically significant, β = -.29, 95% CI [-0.57, -0.01], *p* = .041). However, a significant difference between intervention and wait-list was not observed for change in infertility-related stress specific to relationship concern, need for parenthood, or rejection of childfree lifestyle. Finally, between-group ESs were calculated using the difference in distress outcomes change between groups based on the LCS models and the pooled SD at post-assessment. Between-group ESs favored the intervention over quasi-control wait-list group for depression, anxiety, overall perceived stress, and infertility related social and sexual distress outcomes with medium-to-large effect sizes (*d*s range from .45 to .86; see Table 3).

Due to the substantial differences in time at post-assessment, secondary analyses using the mid-assessment data were performed to determine changes in symptom severity between groups. Both anxiety and depression measures decreased for the intervention group (*MD* = -0.29 and -2.48, respectively) and increased for the quasi-control group (*MD* = 1.12 and 1.54, respectively). The two groups differed significantly for depression (β = -0.25, p = .01), but not for anxiety (β = -0.11, p = .35). For depression, a medium effect size [*d* = 0.61, 39] was found favoring the intervention group [for additional details, see 34]. When examining anxiety scores for those with elevated anxiety (BAI 10 or above), the main effect of the intervention condition on anxiety was significant (β = -0.46, p = .03) and a large effect size favoring the intervention was found (d = 1.07).

The self-reported pregnancy rates at the study close were 20% for the wait-list group and 53% for the intervention group. The median time to pregnancy was 97.21 days (SD = 133.94) for the wait-list group and 78.89 days (SD = 113.06) for the intervention group. Results of a binary logistic regression indicated that the odds of becoming pregnant was 4.47 times higher for participants in the intervention group as compared to the wait-list group, OR 95% CI [1.56, 12.84], *p* = .005. The exploratory survival analysis revealed a significant group difference with the median time (days) until pregnancy for the intervention group being 148.20 days, 95% CI [106.86–189.55], p = .001.

## Discussion

The current pilot study assessed the retention rates, intervention adherence, satisfaction, and secondary outcomes (distress & pregnancy) of an internet-based mind/body program designed for women experiencing infertility. Based on the current methodological developments and findings, participants were willing to engage in, largely adhered to, and were satisfied with the intervention. Furthermore, like the findings in the face-to-face program, participants in the internet-based program showed a reduction in distress and an increase in pregnancy rates as compared to those in the quasi-control wait-list group. The latter findings are qualified by the difference in length between the pre- and post-assessment.

The majority (84%) of participants completed the mid-assessment, and 66% completed the post-assessment. There was a difference in completion rates for the wait-list group versus the intervention group at both assessments, with the wait-list group consistently completing the assessments at a greater rate. The assessment completion rates for the wait-list group were similar or higher than what has been reported in comparable studies; however, the rates for the intervention group were lower than reported in Hämmerli et al. [85%, 16], but similar to what was reported by van Dongen et al. [48%, 17]. For participants randomized to the intervention group, nearly all engaged in at least one module where over half participated in at least five modules. The adherence rates found in this study are comparable to other internet-based studies [16,17].

The intervention group showed significant reduction from baseline to post-assessment for anxious and depressive symptom severity and perceived stress. The quasi-control wait-list group showed a non-significant increase in those same measures of distress across a reporting period shorter on average than the intervention group’s reporting period. The longer length in the intervention group was the result of participants not completing one module per week. The findings in the quasi-control wait-list group are consistent with previous research indicating that distress for women with infertility increases over time [43], while people in therapeutic interventions have shown improvements [20,44]. Effect sizes for the intervention group from pre- to post- and compared to the wait-list post-results ranged from medium to large indicating that distress decreased relatively consistently for those in the intervention group. The secondary analyses at the mid-point assessment, suggested that these changes occurred as early as five weeks. Furthermore, results suggest that pregnancy rates in the intervention group occurred earlier and more frequently as compared to the quasi-control wait-list group. The results seen in the current study are similar to previous findings with interventions using the in-person Mind/Body Program for Fertility [19,21,45].

The current study had several limitations. Only a small proportion of participants completed all ten modules. This suggests that a briefer and/or more tailored program may ultimately be more acceptable to individuals. Although randomization resulted in the intervention and wait-list groups not differing significantly at the pre-assessment on demographic and other variables, the two groups did differ on time between the pre- and post-assessment. This difference in time, resulting from the intervention group requiring substantially more time to complete the modules than initially anticipated, could have influenced the outcome. Based on other data, the flat or declining pattern in the wait-list group would be expected to continue at longer follow-ups that matched the intervention. Given that most participants were recruited online, the results may differ from studies where women are recruited through more traditional means (e.g., reproductive medical practices).

This is one of few studies to examine internet-based interventions for women experiencing infertility. This pilot study successfully recruited and randomized 71 women with important findings emerging. A comprehensive baseline assessment of these participants allowed for the identification of demographic, psychological, and medical characteristics, which could have been controlled for group differences if necessary. The sample was recruited nationally, representing 25 states along with some international representation, and a conservative data analytic approach was used (maximum likelihood estimation with robust standard errors), both of which increase confidence in the findings. Furthermore, improvements in distress ratings relative to a quasi-control group were found despite most participants not completing all ten modules.

Several future research directions are evident from these pilot findings. First, an RCT with groups matched on the pre- to post-assessment length is needed. Further, future trials would benefit from using a structurally equivalent active control condition, such as an online psychoeducation only program, which could better establish efficacy and help isolate specific versus nonspecific therapeutic components of the program. Second, an abbreviated internet-based program for fertility with automated feedback, collection of follow-up data, utilization of multiple e-therapists, inclusion of cost-analyses, and inclusion of data beyond self-report should be conducted. As participants received individualized therapeutic feedback, psychoeducation, and cognitive-behavior tools (i.e., relaxation training, communication strategies) early in the current program, it is possible that these early components may be powerful enough to reduce distress in this population. We believe these findings could inform future adaptive SMART trials that will facilitate the application of precision medicine approaches by identifying treatment response patterns that will allow for innovative strategies in risk identification, prevention, and clinical improvements in personalized care.

An additional direction includes integration of this online intervention within reproductive medical care thus providing built-in support for patients as they go through treatment. It is important to recognize that an internet-based program allows participants to progress at their own rate. While this is a positive, it does potentially extend the time for completion of an intervention.

## Conclusion

The present RCT was the first to assess the potential to translate the face-to-face Mind/Body Program for Fertility into an internet-based intervention and test its acceptability and effectiveness with women experiencing infertility. Satisfaction and intervention adherence suggest feasibility and acceptability. Relative to a quasi-control wait-list group, results suggest improvements may occur in distress ratings and pregnancy rates for women experiencing infertility. Internet-based mind/body interventions in general are a promising area for future research with women (and potentially couples) experiencing infertility.

The current pilot study (a) contributes to the development and design of future research; (b) develops and clarifies hypotheses to be studied; (c) identifies barriers to future study completion (i.e., high rates of participant baseline stress); (d) provides estimates of the expected rates of attrition; (e) reinforces that face-to-face interventions can be translated into acceptable and satisfactory programs for women experiencing infertility; and (f) suggests that, relative to a quasi-control group, decreases in distress and increases in pregnancy rates may occur. These results suggest that the program may have an impact on pregnancy rates. Future research with a larger sample size and more stringent methodological considerations (e.g., consistent timing of assessments per group assignment) are needed to replicate these findings.

As highlighted in Clarke and Currie’s review on the co-morbidity of chronic illness, anxiety, and depression, our current health care model–to either treat the physical disease or the mental illness first–is not effective, efficient, or cost-effective [2]. This is as true for infertility as it is with any other chronic illness. Clarke and Currie suggested that integrated care models are needed, such as a model that includes screening and monitoring as well as psychoeducation regarding the interaction between the disease and mental health, self-management advice and cognitive-behavioral strategies [2]. The findings of the current study suggest that this internet-based mind/body intervention could change the way reproductive medicine is provided. For instance, reproductive medical practices could implement easy and quick screening and monitoring for distressed patients and offer a convenient, effective, and affordable intervention (e.g., internet-based) as an option for these patients, thus providing an increase in emotional support and care.

## Supporting information

S1 Protocol (PDF)

S1 Checklist CONSORT 2010 checklist of information to include when reporting a randomised trial*. (DOC)

S1 File For code, data, and presentations go to: https://doi.org/10.17605/OSF.IO/FC2EJ. (DOCX)
