# Supplementary material for: An internet-based mind/body intervention to mitigate distress in women experiencing infertility: A randomized pilot trial
Source: PLoS One. 2020 Mar 18;15(3):e0229379. doi: 10.1371/journal.pone.0229379 (PMC7080396; doi:10.1371/journal.pone.0229379)
Supplement: S1 Protocol — (PDF) [file pone.0229379.s001.pdf]

Clinicaltrials.org Cover
Study Protocol and Statistical Analysis Plan

Official Title | Fertility & Well-Being: Mind/Body Protocol

NCT Number | Not available

Date of Document | Most recently approved Human Subjects Research Protocol as of 11/08/2017

For Committee Use Only
PROTOCOL NUMBER

Human Subjects Research Protocol

The Common Human Subjects Protocol Cover Form **must** be completed and **attached** to the front of this form. This Protocol form should be completed for any human subjects research proposal that does not have a specific "protocol," such as a grant application. This form must be submitted along with a copy of the complete grant proposal and all the information in this form **must** be consistent with that proposal. This protocol form, once IRB approved, will be the working protocol for that research. **When completing this document, do not refer to page numbers within your grant**. If revisions are necessary during the course of the research, amendments should refer to this protocol form, not the grant proposal. Enter responses for all sections. Check N/A if the section does not apply.

PROTOCOL SUMMARY

Project: Title (Should match the title entered on the face page of any associated grant proposal.)

Fertility & Well-Being: Mind/Body Protocol

Principal Investigator: Jessica Clifton, Ph.D.

Grant Sponsor:

Grant Number:
(For grants routed through UVM, indicate the OSP Proposal ID # located at the top of the OSP Routing Form)

Lay Language Summary: *(Please use non-technical language that would be understood by nonscientific IRB members to summarize the proposed research project. The information must include: (1) a brief statement of the problem and related theory supporting the intent of the study, and (2) a brief but specific description of the procedure(s) involving the human subjects. Please do not exceed one single-spaced 8 ½ X 11" page.)*

A wealth of research, mostly cross-sectional, has corroborated the association between infertility and mood disturbances, including anxiety, stress reactivity, negative affectivity, and depression (e.g. Anderson, Sharpe, Rattray, & Irvine, 2003; K. J. Connolly, Edelmann, & Cooke, 1987; Facchinetti, Volpe, Matteo, Genazzani, & Artini, 1997; Glover, Gannon, Sherr, & Abel, 1996; Greil, 1997; Myers, 1990; L. Schmidt, 2006; Smeenk et al., 2001; Thiering, Beaurepaire, Jones, Saunders, & Tennant, 1993). Compared to the general population, individuals with an infertility diagnosis have been shown to have higher rates of anxiety symptoms (Fassino et al., 2002; Drosdzol & Skrzypulec, 2008). Furthermore, anxiety has been correlated with longer menstrual cycles (e.g., Hjollund et al., 1999), lower pregnancy rates (e.g., Sanders & Bruce, 1997), fewer follicles (e.g., Gurhan, Akyu, Atici, & Kisa, 2009), and fewer live births (e.g., Klonoff-Cohen, Chu, Natarajan, & Sieber, 2001).

The study consists of three-parts:

Part I: The first part of the study will be to assess psychosocial functioning (i.e., psychological symptoms/diagnoses, stress, mindfulness skills, emotion regulation, and relational functioning) and interest in psychological support by women (18 years or older) and their partners (when available) suffering from primary infertility (i.e., never given birth). In addition, the first part of the study will allow us to better understand how psychosocial functioning relates to patient interest in psychological support/treatment. A total of 150 women and their partners (when applicable) will be recruited for the first part of this study. . Based on this we are changing the recruitment process in order to address this issue. We will be inviting fertility clinics, primary care facilities, OBGYN offices, yoga studios, acupuncture offices, and other fertility related health facilities to help recruit for this study by contacting the directors/managers and informing them about the study and how they can get involved (see **Letter to Clinic/Information Sheet**). Specifically, we will be providing clinics with information about the study, along with recruitment flyers to post in their clinic and include in new patient packets (see Recruitment

Flyers) and text to post on their website (when applicable; see text in Recruitment Flyers). Additionally, staff will be asked to inform prospective subjects about the availability of the research and provide prospective subjects with information about the research by providing them with a study flyer with the relevant recruitment information within the new patient packet for infertility patients (when applicable). Individuals will be directed to email the researchers and interested individuals will be given a token specific link that will direct them to the Limesurvey questionnaires. Recruitment will also be conducted through social media sites (e.g., Facebook), websites geared toward infertile couples (e.g., Resolve), crowdsourcing (e.g., Amazon Mechanical Turk), broadcast email (e.g., special interest groups/email lists, such as American Society for Reproductive Medicine), emailing colleagues and acquaintances outside of OBGYN that may know either infertile couples directly or individuals that work with infertile couples, physical/online newspapers (e.g., New York Times, Seven Days), online communities (e.g., Craigslist, Yahoo Classifieds, Front Porch Forum), search engines (e.g., Google advertising, Yahoo), and television interviews when applicable/possible using the "Minimized Recruitment Text."

Part II: The second part of the study will provide an internet-based version of an empirically established mind-body intervention for infertile women. Participants that meet the following criteria will be invited to participate in the second part of the study: (1) interested in participating in online psychological treatment, (2) elevated anxiety symptomology (a score of 8 or higher on the Beck Anxiety Inventory) (3) no current diagnosis of an active psychotic disorder, eating disorder, substance abuse or dependence and not reporting current suicidal ideation or intent (assessed and scored as "high" by the MINI International Neuropsychiatric Interview for DSM-IV), (4) no psychotropic medication changes in the last three months; (5) not currently practicing regular formal relaxation techniques; and (6) not participating in any individual/group psychological treatment. The second part of the study aims to address some of the limitations of prior research by (1) using a manualized, evidence-based treatment tailored for women diagnosed with infertility that has been effective at reducing anxiety and depressive symptoms; (2) including only participants with heightened levels of anxiety symptoms; and (3) including individuals with co-morbid depressive symptoms. In order to fine-tune this novel modality (i.e., internet-based), the first two individuals/couples randomized to the treatment group will be provided with an accelerated version (i.e., two times a week for four weeks) of the treatment in order to obtain feedback and make any necessary adjustments to treatment. This will allow the researchers to fine-tune the intervention protocol/modality so that it is acceptable to participants. Individuals that qualify for the study will be sent a link inviting them to participate in the second part of the study. Participants interested in the study that click on the link will be directed to additional information regarding the study. Individuals who agree to participate will complete pre-treatment assessments and then be randomized between an experimental (i.e., treatment) and a control (i.e., wait-list) condition. A total of 74 participants will be randomized to one of the two groups (i.e., half in treatment, half in wait-list). Participants in the treatment condition will complete minimal assessments weekly (see *Measures*). Participants assigned to the treatment condition will be asked to complete ten weeks of the treatment condition by completing weekly internet-based treatment modules (each estimated to take an hour) and assigned practice exercises during the week (i.e., homework). At the end of the ten weeks all participants invited/qualified for Part II will be asked to complete all post-treatment assessments. Individuals that indicate that they are pregnant, have changed their psychometric medication, begun another mental health treatment, and/or began infertility treatment will be allowed to stay in the intervention, but the data provided by these individuals will be excluded from analyses.

Part III: The third part of this study will assess how baseline psychosocial functioning and group status (i.e., treatment group and/or wait-list group) predicts fertility treatment decision-making and outcome. Based on this, a Limesurvey link will be emailed to all participants 12-months after completion of the study. Participants will be asked to complete a brief survey (10 minutes) answering questions regarding infertility related distress, anxiety, fertility treatment/number of treatment cycles, pregnancy status, decision-making on not pursuing/ending fertility treatment. Endpoints include whether patients continue on to fertility treatment after receiving a diagnosis for infertility and number of treatment cycles before becoming pregnant and/or dropping out of treatment.

PURPOSE AND OBJECTIVES

Purpose: *The importance of the research and the potential knowledge to be gained should be explained in detail. Give background information.*

Infertility is defined as the inability to conceive a child. A couple may be acknowledged as infertile if conception has not occurred after twelve months of having regular, unprotected intercourse (Chandra, Martinez, Mosher, Abma, & Jones, 2005; Jose-Miller & Boyden, 2007). As many as 32% of women are estimated to suffer from infertility (Gurunath et al., 2011). According to the CDC, of the 62 million women in the United States between the ages of 15-44 in 2002, 12% used an infertility service (e.g., advice, medical help to prevent miscarriage, assisted reproductive technology), 1.1% used artificial insemination (e.g., intrauterine insemination) and 0.3% used assisted reproductive technologies (e.g., in vitro fertilization). Assisted reproductive technology (ART) is defined as any fertility treatments where both eggs and sperm are handled (Centers for Disease Control and Prevention, American Society for Reproductive Medicine, Society for Assisted Reproductive Technology, 2009; Implementation of the Fertility Clinic Success Rate and Certification Act of 1992—A Model Program for the Certification of Embryo Laboratories; Notice, 1999). This includes intrauterine insemination (IUI), intracytoplasmic sperm injection (ICSI), gamete intrafallopian transfer (GIFT), zygote intrafallopian transfer (ZIFT), and in vitro fertilization (IVF). In 2007, 142,435 Assisted Reproductive Technology (ART) cycles were performed at 430 reporting clinics in the United States resulting in 57,569 infants (Centers for Disease Control and Prevention, American Society for Reproductive Medicine, Society for Assisted Reproductive Technology, 2009).

Although ART has shown to be highly successful in improving rates of conception research, mostly cross-sectional, has corroborated the association between infertility and mood disturbances, including anxiety, stress reactivity, negative affectivity, and depression (e.g., Anderson, et al., 2003; K. J. Connolly, et al., 1987; Facchinetti, et al., 1997; Glover, et al., 1996; Greil, 1997; Myers, 1990; L. Schmidt, 2006; Smeenk, et al., 2001; Thiering, et al., 1993). In addition, researchers have reported an association between psychosocial factors such as relational functioning (e.g., Carter et al., 2010; Drosdzol & Skrzypulec, 2009; Monga, Alexandrescu, Katz, Stein, & Ganiats, 2004) in regards to infertility diagnosis and infertility treatment. In particular, anxiety has been correlated with longer menstrual cycles (e.g., Hjollund, et al., 1999) lower pregnancy rates (e.g., Sanders & Bruce, 1997), fewer follicles (e.g., Gurhan, et al., 2009), and fewer live births (e.g., Klonoff-Cohen, et al., 2001).

Anxiety is a transitory emotional state that can be characterized by subjective feelings of tension, apprehension, and is accompanied with the activation of the autonomic nervous system and the hypothalamus-pituitary-adrenal axis (e.g. Spielberger, Gorsuch, & Lushene, 1970; Tsigos & Chrousos, 2002). The function of anxiety is to motivate the individual to avoid situations that could be potentially harmful (McNally, 1990; Mowrer, 1939). Because of the protective function of this emotion, being able to experience anxiety and to have an appropriate stress response to a stressor is an important adaptive function for the organism; however, excessive or irrational anxiety can have a number of detrimental effects on people's lives.

Anxiety disorders, which can be characterized as emotional disorders, are prevalent and debilitating psychological disorders that disproportionally affect women (e.g., CDC, 2011) of childbearing age (e.g., Matthey, 2004; Moffitt et al., 2007; Pigott, 2003). Studies estimate that more than 500,000 pregnancies in the United States involve women with psychiatric illnesses that emerged either before or during pregnancy ("ACOG Practice Bulletin," 2008). An estimated 25-30% of infertile women experience heightened anxiety (Chen, Chang, Tsai, & Juang, 2004). Based on the diathesis-stress model (Ingram & Luxton, 2005; Stanton & Dunkel-Schetter, 1991), a major life stressor, such as infertility, which affects the individual on personal, relational, and social levels (Peterson, Gold, & Feingold, 2007), can increase the risk for developing an emotional disorder (i.e., anxiety and/or depressive) even in women with mild vulnerabilities (Verhaak et al., 2005).

Infertility tends to be chronic and the treatment to resolve infertility is stressful, therefore it is not uncommon that these anxiety symptoms are exacerbated as infertility duration increases, with infertility treatment, and even during pregnancy (Austin, Tully, & Parker, 2007; O'Connor, Heron, & Glover, 2002; O'Hara, 2009). Additionally, anxiety symptoms can have detrimental long-term outcomes including inhibiting pregnancy or having detrimental effects on the physical and mental development of the fetus. For instance, high levels of anxiety symptoms during pregnancy are associated with a 2.12-fold increased risk of preeclampsia (Qiu, Williams, Calderon-Margalit, Cripe, & Sorensen, 2009), a condition that leads to reduced blood flow to the uterus that can affect normal development. Animal studies have shown that high levels of anxiety induced by lack of food or resources or lack of safety in the environment are responsible for the alteration of the reproductive cycle in female rats and other mammals (e.g., Baker, Kentner, Konkle, Santa-Maria Barbagallo, & Bielajew, 2006; Keen-Rhinehart et al., 2009; Kondoh et al., 2009). The inhibition of fertility can be useful in situations when resources are scarce and the ability to sustain an offspring are limited but it would have unnecessary repercussions if the anxiety is caused by a maladaptive response to the environment as in the case of individuals with low emotion regulation skills and mindfulness skills.

Studies on anxiety and infertility in humans show that elevated cortisol levels, a hormone involved in the HPA axis, is associated with fewer successful IVF treatments and has been hypothesized to increase uterine contractions thereby obstructing implantation (Fanchin et al., 1998). Also, studies that measured anxiety, either preceding or during treatment (e.g., gonadotrophin-releasing stimulation cycle or prior to the first IVF visit) found an association between anxiety and lower pregnancy rates (Demyttenaere, Nijs, Evers-Kiebooms, & Koninckx, 1992; Facchinetti, et al., 1997; Smeenk, et al., 2001). Anxiety during the early follicular phase and prior to oocyte retrieval was significantly (negatively) associated with β-hCG concentration 15 days after oocyte retrieval and anxiety at the time of oocyte retrieval was also associated with lower prolactin levels 15 days post retrieval and positive pregnancy outcome (Demyttenaere, et al., 1992). Moreover, a study measuring the emotional response to anxiety provoking pictures and the ability of individuals to compete a cognitive task while being exposed to such anxiety provoking stimuli (Stroop Task), thereby testing anxiety activation and regulation. Individuals that, on the evening prior to oocyte retrieval, showed higher reactivity during the anxiety-provoking stimuli, operationlized as greater systolic blood pressure and heart rate, had higher rates of pregnancy failure (Facchinetti, et al., 1997).

Not all studies investigating anxiety and IVF outcome found a significant relationship between the two. Lintsen et al. (2009) failed to find an association between anxiety levels measured one day before oocyte retrieval and pregnancy rates. Similarly, Harlow et al. (1996) reported that greater anxiety during the follicular phase sample as well as on the day of the human chorionic gonadotropin injection was not associated with worse pregnancy outcome. These results suggest that, perhaps, anxiety may not be a crucial factor in the pregnancy success of all women with an infertility problem. Perhaps, individual differences in responses to anxiety-provoking stimuli and in the regulation of anxiety would provide a greater understanding of the characteristics of those women whose fertility is most affected by anxiety and this could lead to tailored interventions that would be more cost effective and show greater efficacy than generalized interventions.

Although the research on infertile women and comorbid anxiety and depression is sparse, the current evidence suggests that the likelihood of comorbidity among these disorders in women experiencing infertility is similar to the general population. In fact, depression symptoms are quite common among infertile women with rates ranging from 10.8% to 86.8% (Chen et al., 2004; Chiaffarino et al., 2011; Demyttenaere et al., 1998; Domar, Broome, Zuttermeister, Seibel, & Friedman, 1992; Drosdzol & Skrzypulec, 2008; Lund, Sejbaek, Christensen, & Schmidt, 2009; Holley, Pasch, Bleil, Gregorich, Katz, Adler, 2015; Nelson, Shindel, Naughton, Ohebshalom, & Mulhall, 2008; Newton, Hearn, & Yuzpe, 1990; Ramezanzadeh et al., 2004; Volgsten et al., 2010, 2008). Similar to studies assessing anxiety symptoms, the large range of rates of depression found in the literature is due to study methodology differences such as specific diagnoses vs. symptom severity. For example, Klemetti and colleagues (2010) reported rates of 11% for any anxiety disorder (i.e., panic disorder, social phobia, agoraphobia, or generalized anxiety disorder) and 8% for any mood disorder (i.e., dysthymia or major depressive disorder) in infertile women whereas Demyttenere and colleagues (1998) reported that 54% of individuals with an infertility diagnosis had mild depressive symptoms and 19% had severe depressive symptoms. In terms of comorbid anxiety and mood disorders, one study of infertile women found that of the 40.2% of women who had a psychiatric disorder, over one-third of these women met criteria for both an anxiety and mood disorder (Chen et al., 2004).

In summary, the literature provides an incomplete picture of the association between anxiety and IVF outcomes. While some studies point to clear theoretical and empirical evidence for an association between anxiety and pregnancy (Demyttenaere, et al., 1992; Facchinetti, et al., 1997; Gurhan, et al., 2009; Sanders & Bruce, 1999; Smeenk, et al., 2001), others have failed to do so (Boivin & Takefman, 1995; Harlow, et al., 1996; Lintsen, et al., 2009; Merari, et al., 2002; Verhaak et al., 2001). Some main limitations in the current literature that prevent a clear understanding of the role of anxiety in IVF outcomes include (1) a lack of a valid and comprehensive measurement of anxiety and (2) the lack of well controlled studies to investigate the causal nature of this relationship. Indeed, all the studies published on this topic have analyzed anxiety levels but have failed to discriminate between individuals with a dysfunctional anxiety patterns as compared to those with more adaptive anxiety responses. Individuals who are susceptible to experience an anxiety disorder show clear patterns of emotional dysregulation that may explain why some studies may have found a significant association while others have failed to do so. To provide an example, for an individual with high emotional dysregulation, anxiety is experienced as an overwhelming and distressing emotion that cannot be modulated and that will result in affecting one's well-being and behavior. Conversely, an individual with high levels of anxiety but with the ability to regulate emotions would be able to cope with the anxiety in a more effective way and this may have a different effect on the outcome. We know from animal studies on exercise that indeed, perception of control is an important component of the stress response since a mouse allowed to run freely on a running wheel will show a reduction of his 'anxiety' (Salam et al., 2009), while

a rat forced to run will show an increase physiological and behavioral responses consistent with anxiety, despite the fact that both rats run the same amount (e.g. Droste et al., 2003; Leasure & Jones, 2008). Since infertility is a cause of stress for all individuals, overall levels of anxiety may not be the principal risk factors but may be only a secondary variable associated with levels of emotional vulnerabilities that tie into the ability of the individual to cope with the stressor.

A second limitation of the literature is the utilization of cross-sectional or prospective studies. While these designs can give us general suggestions on the hypothesized relationships, only an empirical manipulation of the proposed risk factor can provide definitive evidence that emotional vulnerabilities are a risk factor for infertility. One way to manipulate risk factors is by providing patients with a treatment designed to reduce the risk factor and measure whether the reduction in the risk factor also provides a reduction in the targeted outcome. In the case of anxiety and infertility, a few studies have been published on treatments to reduce anxiety and improve pregnancy rates for women with infertility problems. The results of these studies are also mixed, with some studies reporting an improvement in outcome (Domar et al., 1990; Domar, Zuttermeister, et al., 1992; Domar, Clapp, Slawsby, Kessel, et al., 2000) and most studies reporting no effects (Chan, Ng, & Chan, 2006; K. Connolly et al., 1993; De Klerk et al., 2005; Emery et al., 2003; Stewart et al., 1992; Takefman, Brender, Boivin, & Tulandi, 1990). The results for these studies are affected by a multitudes of problems, including the use of non-empirically validated treatments for anxiety, the inclusion of individuals who had no clinical or subclinical levels of anxiety (an anxiety treatment will be ineffective if used with people with no problems with anxiety), and the lack of appropriate control groups. Moreover, in order for the risk factor to be confirmed, the treatment needs to be effective at reducing the targeted emotional vulnerability. Unfortunately, the majority of published studies either reported on reduction in anxiety but failed to report on pregnancy rates associated with reduction of anxiety, or reported on pregnancy outcome and did not report whether the treatment was successful at reducing anxiety.

To date, the Mind-Body intervention (Domar et al., 1990; Domar, Zuttermeister, et al., 1992; Domar, Clapp, Slawsby, Kessel, et al., 2000), which incorporates both mindfulness and cognitive principals (i.e., cognitive restructuring) is the only treatment that has been successful at reducing anxiety and depressive symptoms (Domar et al., 1990; Domar, Zuttermeister, et al., 1992; Domar, Clapp, Slawsby, Kessel, et al., 2000) and increase pregnancy rates (Domar, Clapp, Slawsby, Dusek, et al., 2000; Domar et al., 2011) among women seeking infertility treatment in multiple randomized controlled trials as compared to controls. Although the mind-body intervention shows promise at reducing anxiety and depressive symptoms in this population, more research is needed to assess if this treatment is effective with more impaired individuals. Additionally, some infertile couples are unable or unwilling to access a specialized in-person intervention due to barriers such as fear of being dismissed from infertility treatment, fears of stigmatization, skepticism regarding efficacy, comfort level (i.e., too shy or scared), and difficulties scheduling sessions (Boivin et al., 1999; Wischmann, 2005, 2008). Therefore, alternative modalities should be designed and tested incorporating the promising techniques from the Mind-Body intervention in order to increase availability to those less likely to utilize psychological treatment. One alternative modality would be internet-based interventions that have the potential to address many of these barriers, including increased privacy to reduce perceived stigma, cost effectiveness (as compared to face-to-face therapy), and flexibility to be adapted around the patient's busy schedule. For individuals who live in rural areas or have difficulties accessing mental health care (i.e., individuals that are physically disabled and/or lack of transportation), internet-based interventions are an ideal option to access treatment from a distance.

In order to fill the gap in the literature and determine whether emotional vulnerabilities (i.e., emotional dysregulation and mindfulness) are truly a risk factor for pregnancy outcome we proposed a series of studies designed to test the effects of a mindfulness-based treatment for the reduction of anxiety in women receiving infertility treatment. We have already conducted the first pilot study in this line of research. Although we have not completed all of the analyses in the first pilot study, we were able to (1) successfully recruit 177 women and 91 men over a year, (2) retain 78% of women and 65% of men, and (3) ascertain that nearly 30% of these women had heightened anxiety. After conducting this study, we realized that comorbid depressive symptoms were also quite common and as a result have decided to open up our initial criteria to include participants with comorbid depression. The Mind-Body treatment has shown to be promising at reducing anxiety and depressive symptoms, therefore including individuals that have comorbid depression is appropriate. Based on the success of the initial feasibility study, we have chosen to expand on this initial protocol and incorporate a pilot outcome study. We will translate an already promising in-person Mind-Body treatment protocol (Domar, Clapp, Slawsby, Dusek, et al., 2000; Domar et al., 2011) into an internet-based intervention designed to target anxiety and depressive symptoms. In addition, all data will be analyzed to create a model to predict fertility treatment outcome (failure to begin the

fertility treatment, number of cycles, pregnancy success rates, and drop-outs). We will examine multiple predictors of distress in infertility couples, focusing on variables that may be predictive of fertility treatment outcome.

In summary, the stress of infertility can exacerbate anxiety symptoms in the individual and may play a role in the transmission of anxiety to the child. The proposed intervention will be provided at a very key time of this process: after the individual has developed anxiety and depressive symptoms, but before infertility treatment and/or pregnancy occur. We expect that participants in the treatment group will be motivated to complete the treatment because (1) research has shown that a reduction in anxiety symptoms may facilitate a successful embryo implantation (Domar, Clapp, Slawsby, Dusek, et al., 2000) and (2) treating the anxiety symptoms at this junction may have the beneficial effect of inoculating the individual from the progressive worsening of the symptoms. To test this model, this study will collect data on anxiety and depressive symptoms as well as mindfulness skills.

References*. Include references to prior human or animal research and references that are relevant to the design and conduct of the study.*

ACOG Practice Bulletin: Clinical management guidelines for obstetrician-gynecologists number 92, April 2008 (replaces practice bulletin number 87, November 2007). Use of psychiatric medications during pregnancy and lactation. (2008). Obstetrics and gynecology, 111(4), 1001–1020. doi:10.1097/AOG.0b013e31816fd910

Anderson, K., Sharpe, M., Rattray, A., & Irvine, D. (2003). Distress and concerns in couples referred to a specialist infertility clinic. *Journal of psychosomatic research*, *54*(4), 353–355.

Andersson, G., Carlbring, P., Berger, T., Almlöv, J., & Cuijpers, P. (2009). What makes internet therapy work? *Cognitive behaviour therapy*, *38*(S1), 55–60.

Austin, M.-P., Tully, L., & Parker, G. (2007). Examining the relationship between antenatal anxiety and postnatal depression. *Journal of Affective Disorders*, *101*(1–3), 169–174. doi:10.1016/j.jad.2006.11.015

Baker, S. L., Kentner, A. C., Konkle, A. T., Santa-Maria Barbagallo, L., & Bielajew, C. (2006). Behavioral and physiological effects of chronic mild stress in female rats. *Physiol Behav*, *87*(2), 314–322. doi:10.1016/j.physbeh.2005.10.019

Boivin J, Takefman J, Braverman A. The Fertility Quality of Life (FertiQoL) tool: development and general psychometric properties. *Hum Reprod*uction, 2011;26:2084–91.

Boivin, J., & Takefman, J. E. (1995). Stress level across stages of in vitro fertilization in subsequently pregnant and nonpregnant women. *Fertility and Sterility, 64*(4), 802-810.

Boivin, J., Scanlan, L. C., & Walker, S. M. (1999). Why are infertile patients not using psychosocial counselling? *Human Reproduction*, *14*(5), 1384–1391.

Bunting, L., & Boivin, J. (2008). Knowledge about infertility risk factors, fertility myths and illusory benefits of health habits in young people. *Human Reproduction*, 23 (8), 1858- 186

Carter, J., Raviv, L., Applegarth, L., Ford, J., Josephs, L., Grill, E., Barakat, R. (2010). A cross-sectional study of the psychosexual impact of cancer-related infertility in women: Third-party reproductive assistance. *Journal of Cancer Survivorship, 4*(3), 236-246.

CDC. (2009). Centers for Disease Control and Prevention, American Society for Implementation of the Fertility Clinic Success Rate and Certification Act of 1992—A Model Program for the Certification of Embryo Laboratories; Notice. (1999). (Pub. L. 102–493, 42 U.S.C. 263a–1 et seq.).

CDC. (2011). Mental Illness Surveillance Among Adults in the United States. Centers for Disease Control and Prevention. Retrieved from http://www.cdc.gov/mmwr/preview/mmwrhtml/su6003a1.htm

Center for Disease Control and Prevention. (2011, December, 1). How much physical activity do adults need? Retrieved from http://www.cdc.gov/physicalactivity/everyone/guidelines/adults.html#Musclestrengthening

Chan, C., & Ng, E. (2006). Effectiveness of psychosocial group intervention for reducing anxiety in women undergoing in vitro fertilization: a randomized controlled study. *Fertility and Sterility*, *85*(2), 339–346.

Chandra, A., Martinez, G. M., Mosher, W. D., Abma, J. C., & Jones, J. (2005). Fertility, family planning, and reproductive health of U.S. women: Data from the 2002 National Survey of Family Growth. (U. S. D. of H. and H. Services, Ed.). U.S. Government Printing Office. Retrieved from http://www.cdc.gov/nchs/data/series/sr_23/sr23_025.pdf

Chen, T. H., Chang, S. P., Tsai, C. F., & Juang, K. D. (2004). Prevalence of depressive and anxiety disorders in an assisted reproductive technique clinic. *Human Reproduction*, *19*(10), 2313.

Chen, T. H., Chang, S. P., Tsai, C. F., & Juang, K. D. (2004). Prevalence of depressive and anxiety disorders in an assisted reproductive technique clinic. Human Reproduction, 19(10), 2313.

Chiaffarino, F., Baldini, M. P., Scarduelli, C., Bommarito, F., Ambrosio, S., D'Orsi, C., Torretta, R., Bonizzoni, M.Ragni, G. (2011). Prevalence and incidence of depressive and anxious symptoms in couples undergoing assisted reproductive treatment in an Italian infertility department. European Journal of Obstetrics, Gynecology, and Reproductive Biology, 158(2), 235–241. doi:10.1016/j.ejogrb.2011.04.032

Connolly, K., Edelmann, R., & Cooke, I. (1987). Distress and marital problems associated with infertility. *Journal of Reproductive and Infant Psychology*, *5*(1), 49–57.

Connolly, K., Edelmann, R., Bartlett, H., Cooke, I., Lenton, E., & Pike, S. (1993). Counselling: An evaluation of counselling for couples undergoing treatment for in-vitro fertilization. *Human Reproduction*, *8*(8), 1332

De Klerk, C., Hunfeld, J., Duivenvoorden, H., Den Outer, M., Fauser, B., Passchier, J., & Macklon, N. (2005). Effectiveness of a psychosocial counselling intervention for first-time IVF couples: a randomized controlled trial. Human Reproduction, 20(5), 1333.

Demyttenaere, K., Bonte, L., Gheldof, M., Vervaeke, M., Meuleman, C., Vanderschuerem, D., & D'Hooghe, T. (1998). Coping style and depression level influence outcome in in vitro fertilization. Fertility and Sterility, 69(6), 1026–1033.

Demyttenaere, K., Nijs, P., Evers-Kiebooms, G., & Koninckx, P. (1992). Coping and the ineffectiveness of coping influence the outcome of in vitro fertilization through stress responses. *Psychoneuroendocrinology*, *17*(6), 655–665. doi:10.1016/0306-4530(92)90024-2

Derogatis, L. R. (1975). *Brief Symptom Inventory*. Baltimore, MD: Clinical Psychometric Research.

Derogatis, L. R., & Melisaratos, N. (1983). The Brief Symptom Inventory: an introductory report. *Psychological Medicine*, 13(3), 595-605.

Domar, A. D., Broome, A., Zuttermeister, P. C., Seibel, M., & Friedman, R. (1992). The prevalence and predictability of depression in infertile women. Fertility and Sterility, 58(6), 1158.

Domar, A. D., Clapp, D., Slawsby, E., Kessel, B., Orav, J., & Freizinger, M. (2000). The impact of a group psychological intervention on distress on distress in infertile women. Health Psychology, 19(6): 568-75.

Domar, A. D., Rooney, K. L., Wiegand, B., Orav, E. J., Alper, M. M., Berger, B. M., & Nikolovski, J. (2011). Impact of a group mind/body intervention on pregnancy rates in IVF patients. *Fertility and Sterility*, *95*(7), 2269–2273. doi:10.1016/j.fertnstert.2011.03.046

Domar, A. D., Seibel, M., & Benson, H. (1990). The mind/body program for infertility: a new behavioral treatment approach for women with infertility. *Fertility and Sterility*, *53*(2), 246.

Domar, A. D., Zuttermeister, P. C., Seibel, M., & Benson, H. (1992). Psychological improvement in infertile women after behavioral treatment: a replication. *Fertility and sterility*, *58*(1), 144–147.

Drosdzol, A., & Skrzypulec, V. (2008). Quality of life and sexual functioning of Polish infertile couples. *The European Journal of Contraception and Reproductive Health Care*, *13*(3), 271–281.

Drosdzol, A., & Skrzypulec, V. (2008). Quality of life and sexual functioning of Polish infertile couples. The European Journal of Contraception and Reproductive Health Care, 13(3), 271–281.

Drosdzol, A., & Skrzypulec, V. (2009). Evaluation of Marital and Sexual Interactions of Polish Infertile Couples. *The Journal of Sexual Medicine, 6*(12), 3335-3346. doi: 10.1111/j.1743-6109.2009.01355.x

Droste, S. K., Gesing, A., Ulbricht, S., Müller, M. B., Linthorst, A. C. E., & Reul, J. M. H. M. (2003). Effects of Long-Term Voluntary Exercise on the Mouse Hypothalamic-Pituitary-Adrenocortical Axis. *Endocrinology, 144*(7), 3012-3023. doi: 10.1210/en.2003-0097

Emery, M., Béran, M., Darwiche, J., Oppizzi, L., Joris, V., Capel, R., … Germond, M. (2003). Results from a prospective, randomized, controlled study evaluating the acceptability and effects of routine pre IVF counselling. *Human Reproduction*, *18*(12), 2647.

Facchinetti, F., Volpe, A., Matteo, M., Genazzani, A., & Artini, G. (1997). An increased vulnerability to stress is associated with a poor outcome of in vitro fertilization-embryo transfer treatment. *Fertility and Sterility*, *67*(2), 309–314.

Fanchin, R., Righini, C., Olivennes, F., Taylor, S., de Ziegler, D., & Frydman, R. (1998). Uterine contractions at the time of embryo transfer alter pregnancy rates after in-vitro fertilization. *Hum Reprod*, *13*(7), 1968–1974.

Fassino, S., Piero, A., Boggio, S., Piccioni, V., & Garzaro, L. (2002). Anxiety, depression and anger suppression in infertile couples: a controlled study. *Human Reproduction*, *17*(11), 2986.

Frequently Asked Questions About the Mind/Body Programs at the Domar Center. (2013). Retrieved September 19, 2013, from https://www.domarcenter.com/services/programs/programs_faq.html

Glover, L., Gannon, K., Sherr, L., & Abel, P. D. (1996). Distress in sub-fertile men: A longitudinal study. *Journal of Reproductive and Infant Psychology*, *14*(1), 23–36.

Greil, A. L. (1997). Infertility and psychological distress: A critical review of the literature. *Social Science & Medicine*, *45*(11), 1679–1704. doi:10.1016/s0277-9536(97)00102-0

Gurhan, N., Akyu, A., Atici, D., & Kisa, S. (2009). Association of Depression and Anxiety with Oocyte and Sperm Numbers and Pregnancy Outcomes During in Vitro Fertilization Treatment. *Psychological Reports*(104), 10.

Gurunath, S., Pandian, Z., Anderson, R. A., & Bhattacharya, S. (2011). Defining infertility--a systematic review of prevalence studies. *Human reproduction update*, *17*(5), 575–588. doi:10.1093/humupd/dmr015

Harlow, C., Fahy, U., Talbot, W., Wardle, P., & Hull, M. (1996). Stress and stress-related hormones during in-vitro fertilization treatment. *Human Reproduction, 11*(2), 274.

Hjollund, N. H., Jensen, T. K., Bonde, J. P. E., Henriksen, T. B., Andersson, A., Kolstad, H. A., Olsen, J. (1999). Distress and reduced fertility: a follow-up study of first-pregnancy planners. *Fertility and Sterility, 72*(1), 47-53. doi: 10.1016/s0015-0282(99)00186-7

Holley, S. R., Pasch, L. A., Bleil, M. E., Gregorich, S., Katz, P. K., & Adler, N. E. (2015). Prevalence and predictors of major depressive disorder for fertility treatment patients and their partners. Fertility and Sterility, 103(5), 1332-1339. doi: 10.1016/j.fertnstert.2015.02.018

Ingram, R. E., & Luxton, D. D. (2005). Vulnerability-stress models. *Development of psychopathology: A vulnerability-stress*

perspective, 32–46.
Jose-Miller, A. B., & Boyden, J. W. (2007). Infertility. *American family physician, 75*, 849-858.
Keen-Rhinehart, E., Michopoulos, V., Toufexis, D., Martin, E., Nair, H., Ressler, K., Wilson, M. (2009). Continuous expression of corticotropin-releasing factor in the central nucleus of the amygdala emulates the dysregulation of the stress and reproductive axes. *Molecular Psychiatry*, *14*(1), 37–50. doi:10.1038/mp.2008.91
Klemetti, R., Raitanen, J., Sihvo, S., Saarni, S., & Koponen, P. (2010). Infertility, mental disorders and well-being–a nationwide survey. Acta obstetricia et gynecologica Scandinavica, 89(5), 677–682.
Klonoff-Cohen, H., Chu, E., Natarajan, L., & Sieber, W. (2001). A prospective study of stress among women undergoing in vitro fertilization or gamete intrafallopian transfer* 1. *Fertility and Sterility, 76*(4), 675-687.
Kondoh, E., Okamoto, T., Higuchi, T., Tatsumi, K., Baba, T., Murphy, S., Fujii, S. (2009). Stress affects uterine receptivity through an ovarian-independent pathway. *Human Reproduction*, *24*(4), 945.
Leasure, J. L., & Jones, M. (2008). Forced and voluntary exercise differentially affect brain and behavior. *Neuroscience, 156*(3), 456-465. doi: 10.1016/j.neuroscience.2008.07.041
Lintsen, A., Verhaak, C., Eijkemans, M., Smeenk, J., & Braat, D. (2009). Anxiety and depression have no influence on the cancellation and pregnancy rates of a first IVF or ICSI treatment. *Human Reproduction*.
Lund, R., Sejbaek, C., Christensen, U., & Schmidt, L. (2009). The impact of social relations on the incidence of severe depressive symptoms among infertile women and men. Human Reproduction, 24(11), 2810.
Manhal-Baugus, M. (2001). E-therapy: practical, ethical, and legal issues. *Cyberpsychology & behavior: the impact of the Internet, multimedia and virtual reality on behavior and society*, *4*(5), 551–563.
Matthey, S. (2004). Detection and treatment of postnatal depression (perinatal depression or anxiety). *Current Opinion in Psychiatry*, *17*(1), 21–29.
McNally, R. J. (1990). Psychological approaches to panic disorder: a review. *Psychol Bull*, *108*(3), 403–419.
Merari, D., Chetrit, A., & Modan, B. (2002). Emotional reactions and attitudes prior to in vitro fertilization: an inter-spouse study. *Psychology & Health, 17*(5), 629-640.
Moffitt, T., Harrington, H., Caspi, A., Kim-Cohen, J., Goldberg, D., Gregory, A., & Poulton, R. (2007). Depression and generalized anxiety disorder: Cumulative and sequential comorbidity in a birth cohort followed prospectively to age 32 years. *Archives of General Psychiatry*, *64*(6), 651–660. doi:10.1001/archpsyc.64.6.651
Monga, M., Alexandrescu, B., Katz, S. E., Stein, M., & Ganiats, T. (2004). Impact of infertility on quality of life, marital adjustment, and sexual function. *Urology, 63*(1), 126-130. doi: 10.1016/j.urology.2003.09.015
Mowrer, O. H. (1939). A stimulus-response analysis of anxiety and its role as a reinforcing agent. *Psychological Review*, *46*, 553–565.
Myers, M. F. (1990). Male gender-realted issues in reproductive technology. In I. N. Stotland (Ed.), *Psychiatric aspects of reproductive technology* (pp. 25–35). Washington, DC: American Psychiatric Press.
Nelson, C., Shindel, A., Naughton, C., Ohebshalom, M., & Mulhall, J. (2008). Prevalence and predictors of sexual problems, relationship stress, and depression in female partners of infertile couples. Journal of Sexual Medicine, 5(8), 1907–1914.
Newton, C. R., Hearn, M. T., & Yuzpe, A. A. (1990). Psychological assessment and follow-up after in vitro fertilization: Assessing the impact of failure. Fertility and Sterility, 54(5), 879–886.
O'Connor, T. G., Heron, J., & Glover, V. (2002). Antenatal anxiety predicts child behavioral/emotional problems independently of postnatal depression. *Journal of the American Academy of Child & Adolescent Psychiatry*.
O'Hara, M. W. (2009). Postpartum depression: what we know. *Journal of Clinical Psychology*, *65*(12), 1258–1269. doi:10.1002/jclp.20644
Peterson, B., Newton, C., & Feingold, T. (2007). Anxiety and sexual stress in men and women undergoing infertility treatment. *Fertility and Sterility*, *88*(4), 911–914.
Pigott, T. A. (2003). Anxiety disorders in women. *The Psychiatric clinics of North America*, *26*(3), 621–672, vi–vii.
Qiu, C., Williams, M. A., Calderon-Margalit, R., Cripe, S. M., & Sorensen, T. K. (2009). Preeclampsia risk in relation to maternal mood and anxiety disorders diagnosed before or during early pregnancy. *American journal of hypertension*, *22*(4), 397–402. doi:10.1038/ajh.2008.366
Ramezanzadeh, F., Aghssa, M., Abedinia, N., Zayeri, F., Khanafshar, N., Shariat, M., & Jafarabadi, M. (2004). A survey of relationship between anxiety, depression and duration of infertility. BMC Women's Health, 4(1), 9-16.
Reproductive Medicine, Society for Assisted Reproductive Technology. Centers for Disease Control and Prevention. Retrieved from http://www.cdc.gov/art/
Rounsaville, B. J., Carroll, K. M., & Onken, L. S. (2001). A stage model of behavioral therapies research: Getting started and moving on from stage I. Clinical Psychology: Science and Practice, 8(2), 133–142.
Sanders, K., & Bruce, N. (1997). A prospective study of psychosocial stress and fertility in women. *Human Reproduction, 12*(10), 2324-2329. doi: 10.1093/humrep/12.10.2324
Sanders, K., & Bruce, N. (1999). Psychosocial stress and treatment outcome following assisted reproductive technology. *Human Reproduction, 14*(6), 1656.
Schmidt, L. (2006). Psychosocial burden of infertility and assisted reproduction. *Lancet*, *367*(9508), 379–380. doi:10.1016/s0140-6736(06)68117-8

Smeenk, J., Verhaak, C., Eugster, A., Van Minnen, A., Zielhuis, G., & Braat, D. (2001). The effect of anxiety and depression on the outcome of in-vitro fertilization. *Human Reproduction*, *16*(7), 1420.
Spielberger, C. D., Gorsuch, R. I., & Lushene, R. E. (1970). *The STAI Manual for the State-Trait Anxiety Inventory*. Palo Alto, C.A.: Consulting Psychologists Press.
Stanton, A., & Dunkel-Schetter, C. (1991). Psychological adjustment to infertility. *Infertility: Perspectives from stress and coping research*, 3–16.
Stewart, D., Boydell, K., McCarthy, K., Swerdlyk, S., Redmond, C., & Cohrs, W. (1992). A prospective study of the effectiveness of brief professionally-led support groups for infertility patients. *The International Journal of Psychiatry in Medicine*, *22*(2), 173–182.
Takefman, J., Brender, W., Boivin, J., & Tulandi, T. (1990). Sexual and emotional adjustment of couples undergoing infertility investigation and the effectiveness of preparatory information. *Journal of Psychosomatic Obstetrics & Gynecology, 11*(4), 275-290. doi: doi:10.3109/01674829009084423
Thiering, P., Beaurepaire, J., Jones, M., Saunders, D., & Tennant, C. (1993). Mood state as a predictor of treatment outcome after in vitro fertilization/embryo transfer technology (IVF/ET). *Journal of psychosomatic research*, *37*(5), 481–491.
Tsigos, C., & Chrousos, G. P. (2002). Hypothalamic-pituitary-adrenal axis, neuroendocrine factors and stress. *Journal of psychosomatic research*, *53*(4), 865–871. doi:10.1016/s0022-3999(02)00429-4
Verhaak, C., Smeenk, J., Eugster, A., van Minnen, A., Kremer, J., & Kraaimaat, F. (2001). Stress and marital satisfaction among women before and after their first cycle of in vitro fertilization and intracytoplasmic sperm injection* 1. *Fertility and Sterility*, *76*(3), 525–531.
Verhaak, C., Smeenk, J., Evers, A., van Minnen, A., Kremer, J., & Kraaimaat, F. (2005). Predicting emotional response to unsuccessful fertility treatment: a prospective study. *Journal of behavioral medicine*, *28*(2), 181–190.
Volgsten, H., Skoog Svanberg, A., Ekselius, L., Lundkvist, Ö., & Sundström Poromaa, I. (2010). Risk factors for psychiatric disorders in infertile women and men undergoing in vitro fertilization treatment. Fertility and Sterility, 93(4), 1088–1096.
Wischmann, T. (2005). [Psychosocial aspects of fertility disorders]. *Der Urologe. Ausg. A*, *44*(2), 185–194; quiz 195.
Wischmann, T. (2008). Implications of psychosocial support in infertility-a critical appraisal 1. *Journal of Psychosomatic Obstetrics & Gynecology*, *29*(2), 83–90.

Objectives: *Clearly state the primary and secondary objective(s) of the study.*

Summary of Goals:

1. To evaluate patients willingness to be recruited for the intervention and randomized to either the treatment or wait-list group
2. To evaluate patients' adherence and attrition by assessing completion and satisfaction of the intervention
3. To evaluate anxiety and depressive symptoms reduction as well as enhancement of mindfulness and emotion regulation skills.
4. To identify and explore the influence of psychological, relational and emotional functioning, and partners psychosocial functioning on treatment discontinuation and success rates of treatment cycles.

METHODS AND PROCEDURES

Study Design: *Describe the research design, including a description of any new methodology and its advantage over existing methodologies.*

This is a 3-part pilot study that will use both a cross-sectional and longitudinal design.

Part I: A cross-sectional design. Participants will be asked to answer online questionnaires. Consent and the completion of these questionnaires are expected to take approximately **60 to 90 minutes**.

Part II: A between groups repeated measure experimental design. Participants that meet inclusion criteria and are interested in continuing with the study will be randomized to either the treatment or wait-list group. Participants randomized to the treatment group will be provided with 10 online treatment modules provided weekly over 10 weeks. After 10-weeks the wait-list group will have the potential to participate in the treatment protocol if they desire. All participants will be asked to complete pre-, mid-, and post- treatment questionnaires.

Part III: A brief survey will be provided to all participants that completed Part I of the study an estimated 12 months after the completion of Part I. This questionnaire is expected to take approximately **15 minutes**.

Procedures: *Describe all procedures (sequentially) to which human participants will be subjected. Identify all procedures that are considered experimental and/or procedures performed exclusively for research purposes. Describe the types, frequency and duration of tests, study visits, interviews, questionnaires, etc. Include required screening procedures performed before*

enrollment and while on study. Please provide in table, list or outline format for ease of review. (describe and attach all instruments)

Note: A clinical research protocol may involve interventions that are strictly experimental or it may involve some aspect of research (e.g., randomization among standard treatments for collection and analysis of routine clinical data for research purposes). It is important for this section to distinguish between interventions that are experimental and/or carried out for research purposes versus those procedures that are considered standard therapy. In addition, routine procedures performed solely for research purposes (e.g., additional diagnostic/follow-up tests) should be identified.

Pre(Part I): MTurk Only

- Online Assessment (~2 minutes)

Part I: (~ 60 minutes)

- Online Compensation
- Online Assessment (~60-90 minutes)
- Online Directions to Part II of the Study (for interested/qualified participants)

Part II: (~ 670 minutes)

- Pre-treatment questionnaires (~10 minutes)
- Randomization (wait-list/treatment group)
- Pre-treatment compensation (wait-list group only)
- 10 Mind/Body Treatment Modules w/ ongoing questionnaires (~ 30-60 minutes per module)
- Mid-treatment questionnaires (~ 15 minutes)
- Post-treatment questionnaires (~ 30 minutes)
- Post-treatment compensation (wait-list group only)
- Wait-list group offered opportunity to complete 10 Mind/Body Treatment Modules w/ Pre- Mid- and Post-Assessment

Part III: (~15 minutes)

- Online Assessment (~15 minutes/12 months after completing Part I)

Pre-Part I

A total of 350 women will be recruited via MTurk to complete part of the already approved online survey. Specifically, participants will be provided information about the entire study and asked the following questions: (1) age; (2) relationship status; (3) are you an individual trying to get physically pregnant/ are you the partner of someone trying to get physically pregnant/I am not trying to get pregnant at this time; (4) how long have you been trying to get pregnant; (5) zipcode; (6) information on the Online Mind/body Fertility Program summary and would you be interested in participating in the online mind body treatment program. After participants complete the questions, they will be thanked for their participation and asked to email onlinemindbody@uvm.edu if they would like to participate in the rest of the study.

Participants will be recruited from and compensated via MTurk, but their responses will not be connected in any way to their MTurk accounts. This confidentiality, which will make it impossible to identify individual participants, will be accomplished by giving participants a link on MTurk to Limesurvey. After the completion of the brief screener, they will be given a completion code to enter into MTurk. That code is not associated or stored with the participant's Limesurvey screener and will not be stored or retained by Limesruvey in any way, and can thus not be used to associate MTurk account information with survey responses. It is thus impossible to identify any participant's responses.

After participants complete the screener (the survey is anticipated to take 2 minutes or less). Participants who

complete the screener will be compensated $0.10.

Part I

Recruitment

We will be inviting fertility clinics, primary care facilities, OBGYN offices, yoga studios, acupuncture offices, and other fertility related health facilities to help us in recruiting for this study by contacting the directors/managers of each of the clinics and informing them about the study and how they can get involved (see **Letter to Clinic/Information Sheet**). Specifically, we will be providing clinics with information about the study, along with recruitment flyers to post in their clinic and include in new patient packets (see **Recruitment Flyer**) and text to post on their website (when applicable; see text in Recruitment Flyers). Additionally, staff will be asked to inform prospective subjects about the availability of the study by providing them with a study flyer with the relevant recruitment information within the new patient packets for infertility patients (when applicable). All screening will be conducted online. Individuals will be directed to email the researchers and interested individuals will be given a token specific link that will direct them to the Limesurvey questionnaires. Individuals that complete the questionnaire will be asked to inform their partners to contact us by email if they are interested and to provide their partners study ID. Up to 300 women and potentially their partners will be recruited for this part of the study. Additionally, to assess the effectiveness of our recruitment strategy we will be asking fertility clinics the number of fertility patients they saw at their clinics and will be recording the information when available. Recruitment will also be conducted through social media sites (e.g., Facebook), websites geared toward infertile couples (e.g., Resolve), crowdsourcing (e.g., Amazon Mechanical Turk), broadcast email (e.g., special interest groups/email lists, such as American Society for Reproductive Medicine), emailing colleagues and acquaintances outside of OBGYN that may know either rinfertile couples directly or individuals that work with infertile couples, physical/online newspapers (e.g., New York Times, Seven Days), online communities (e.g., Craigslist, Yahoo Classifieds, Front Porch Forum), search engines (e.g., Google advertising, Yahoo), and television interviews when applicable/possible using the “Minimized Recruitment Text.”

Consenting: Informed Consent

Information about the study will be provided at the beginning of the Part I Limesurvey questionnaire. Separate Research Information will be provided to each of the partners in order to clarify the role of each person in the couple. Participants that agree to participate will be asked to give their consent by selecting “yes” (see **Research Information Script, Research Information Script for Partners** and **Request for Waiver of Informed Consent/Authorization/Documentation**). Participants will be encouraged to ask questions or raise concerns prior to consenting and will be given the option to click “I have questions/concerns” and provided with a comment box. A researcher will contact the individual by email to address the questions/concerns. Participants that are still interested after their questions/concerns are addressed will be redirected to the token-specific link and requested to select “yes” indicating their consent.

Psychological Referrals

All participants that consent to Part I will be provided with psychological referrals in case they are interested in seeking psychological care/support and to provide them with emergency contact information (see **Psychological Referral Form**).

Debriefing

At the end of Part I the participants will be thanked for their time and informed that within one week the study coordinator will let them know whether they qualify for Part II of the study. If the participant qualifies for Part II of the study they will be contacted by the research coordinator by email with a reminder about Part II of the study and a link on how to begin the pre-treatment assessment (see **Online Instructions/Emails)**.

Psychosocial Assessments (see section below on questionnaires)

Participants will be given a token specific Limesurvey link that will connect each participants with a unique identification key to link to a protected server where they can complete the online surveys (see section on **Data Safety and Confidentiality**). We will record participants name, partners name and involvement (when applicable) email address and unique study ID in a password protected document only accessible to the study coordinator. This will allow the study coordinator to link the participants with the multiple surveys as well as to their partners (see **Confidentiality** Section for more information). In order to track and connect partners, they will be given the same study IDs and for each a letter will be attached by the ID (i.e., 1A = female participant and 1B = male partner participant). Tracking the contact information will allow us the ability to remind participants to complete questionnaires and/or treatment modules; follow-up with participants whom identify themselves as a risk to self/other and provide them through email emergency contact numbers; and link the questionnaires.

The questionnaires involved in Part I are projected to take between 60 and 90 minutes to complete. Questionnaires will include comprehensive demographic questions that includes variables about gender, sexual orientation, ethnicity, education, work/employment, yearly household income, insurance, thoughts about psychological treatment and marital status. Following these initial questions, subjects will be administered a series of questionnaires asking about psychological symptoms, emotional regulation, and relational adjustment (a complete list and description of each of the questionnaires is provided below). Participants will have the opportunity during this part of the study to decline participating in the second part of the study.

Participants that do not qualify for Part II of the study will be informed and reminded of Part III of the study.

Compensation

Participants will be entered in a drawing to receive a $25 Amazon e-gift card. They will have a 1 in 25 chance of receiving a e-gift card.

Part II

Recruitment

A total of 74 eligible participants will be invited to participate in the second part of the study. Twenty will be randomized to the Treatment Group and twenty to the Wait-List Group.

Consenting

A study coordinator will contact participants that meet eligibility criteria for Part II of the study to inform them by email about their eligibility for Part II. Additionally, within the same email, participants that are eligible will be provided with additional information (see **Online Instructions/Emails)** and a token-specific link to complete the pre-treatment assessments. At the beginning of the pre-treatment assessment will be the information that was provided in the consent form they signed and participants will be asked to re-consent by checking Yes or No on the online survey. After participants complete the pre-treatment assessment they will be contacted by email with their group assignment and the online assessment schedule.

Psychological Assessment (see section below on questionnaires)

The questionnaires for Part II of the study are projected to take an estimated 55 minutes to complete given three times over 12-weeks. Participants in both the wait-list and treatment group will be asked to complete online pre-treatment assessments, mid-treatment assessments (approximately five weeks after the completing the pre-treatment assessment) and post-treatment assessments (approximately 10 weeks after the pre-treatment assessment).

Randomization

Randomization will be determined by using a random number generator via Random.org. This list will be pre-generated by the research coordinator, and this researcher will be responsible for indicating participant randomization group status. Participants will be evenly distributed across groups so that twenty participants will be in each condition. Participants will be informed during the consent process that they will have equal odds of being assigned to one of two groups (i.e., treatment or wait-list). Participants will be informed at the end of the pre-treatment assessment that they will be receiving an email shortly (within two days) indicating their group status and further directions. Participants that are randomized into the treatment group will receive an email with their group status and directions on how to complete the first treatment module.

Treatment

Participants randomized to the experimental condition will receive an empirically supported mind-body training (Domar et al., 1990; Domar, Zuttermeister, et al., 1992; Domar, Clapp, Slawsby, Kessel, et al., 2000) that will be translated into an internet-based treatment (see **Mind-Body Protocol** for more details on content). The mind-body program was originally delivered in a group format one time a week over 10 weeks (they will be given a max of 12 weeks to complete). The information and exercises will be structured into ten different modules using **LimeSurvey**. Each module will be provided weekly. In general, each module will include similar components, including: assessment (i.e., BAI and BDI), quiz on information already learned, relaxation exercise (i.e., meditation, yoga, mindfulness, and deep breathing), opportunity to journal about the stressors of the week and/or progress of homework (i.e., relaxation/mindfulness exercises), psychoeducation (i.e., regarding stress response, nutrition), “mini” relaxation-response exercise, open-responses for reflections, as well as quizzes to assess knowledge gained. At the end of each treatment module participants will be asked to comment on their ability to complete the module, including ease (0 ‘easy’, 1 ‘moderately challenging’, 2 ‘challenging’) and helpfulness of module (0 ‘completely unhelpful’, 1 ‘slightly helpful’, 2 ‘moderately helpful’, 3 ‘very helpful’).

Each week participants will be sent an email that will include (1) positive remarks on their completion of the treatment module, (2) individualized symptom summary, and (3) information on when the next treatment module that will be available. A treatment module will be released every seven days. After the first treatment module, participants will be provided with an individualized summary of the trajectory of their symptoms change (as measured by the BAI) over the course of treatment.

The ten treatment modules include: (1) psychoeducation regarding the physiology of stress, the relaxation response, and the relationship between stress and the reproductive system; (2) diaphragmatic breathing, mini relaxation exercises, learning effective communication; (3) psychoeducation surrounding self-nurturance; (4) psychoeducation surrounding impact of lifestyle behavior on infertility; (5) evaluating the pros and cons of exercises and introduction to hatha yoga; (6) psychoeducation surrounding stress management and cognitive restructuring (i.e., the confrontation and subsequent rethinking of recurrent negative thought patterns); (7) hatha yoga, reducing stress with humor, life road map, psychoeducation surrounding listening/communication; (8) continue cognitive restructuring; (9) provide partner with information regarding support, stories regarding mind/body participants who have chosen adoption or egg donation, psychoeducation surrounding emotional expression, coping and effectively expressing anger; (10) assertiveness training, goal setting, and a summary/review of the program.

Internet-based interventions designed to address panic disorder, social anxiety disorder, posttraumatic stress disorder, mild to moderate depression, and headache could be considered empirically supported (Andersson et al., 2009). In general, web-based interventions have several benefits including accessing hard-to-reach populations, convenience, and cost-effectiveness (Manhal-Baugus, 2001). For example, the internet can reach people who choose not to attend face-to-face treatment or are without many options to treatment providers, such as those who live in rural areas. Furthermore, internet-based interventions can be extremely convenient for those who have scheduling conflicts, difficulties with transportation, or for those with disabilities. Lastly, once developed, internet-based interventions are extremely easy to implement and tend to be extremely affordable when compared to face-to-face therapy.

After participants in the wait-list group complete the post-treatment assessment they will be provided the opportunity to complete the mind-body treatment modules regardless of meeting study criteria at that time (i.e. pregnant, change in medications).

Mini Pilot Testing of the Online Protocol

The first two participants randomized into the Treatment Group will be provided with a modified treatment schedule in order to pilot the online protocol. These first two participants will be asked to complete two modules a week over 4 weeks. These two participants (or four if their partners choose to participate) will be asked to complete the mid-treatment (2-weeks into the treatment) and post-treatment assessments (at the end of treatment) at a faster rate as well. This will provide the researchers with information regarding the usability and functionality of the online modules. Participants will be asked at the end of each of the modules what they liked and didn't like about the treatment protocol and/or look, feel, and function of the online program. This will provide us with information to improve the protocol prior to running all participants through the protocol.

Compensation

Control participants will receive $10 Amazon e-gift certificate for completing the initial online pre-treatment questionnaire and $15 Amazon e-gift certificate upon completion of the online post-treatment questionnaires as an incentive to complete the study. Similar compensation has been noted in other RCT's with the mind-body intervention (Domar et al., 2011). Since the mind-body intervention currently is offered as an in-person group format commercially for $780 ("FAQ's," 2013), participants randomized to the treatment group will receive the treatment itself as compensation.

Part III

A Limesurvey link will be emailed to all participants 12-months after completion of the study. Participants will be asked to complete a brief survey (15 minutes) answering questions regarding infertility related distress, anxiety, depression, fertility treatment/number of treatment cycles, pregnancy status, decision-making on not pursuing/ending fertility treatment.

General

Contacting Participants

Participants will be informed during consenting that they will be contacted using the contact information they provide researchers through email (1) to remind participants to complete questionnaires/treatment modules; and (2) to contact participants if they report thoughts of hurting themselves or others and they will be emailed emergency contact information. All participants will be provided with psychological referrals and emergency contact numbers at the end of Part I of the survey. Additionally, feedback provided during Part II of the study will be provided through email.

For the purpose of maintaining data integrity, for surveys that are incomplete (i.e., missing answers) an online survey with the specific question/measurements will be emailed to participants. Participants will be asked to complete questions they may have mistakenly skipped during the initial survey. They will be provided with the option to do nothing if they intentionally did not fill out the measurement in question (see **Online Instructions/Emails**).

For research involving survey, questionnaires, etc.: *Describe the setting and the mode of administering the instrument and the provisions for maintaining privacy and confidentiality. Include the duration, intervals of administration, and overall length of participation. (describe and attach all instruments)*

☐ ***Not applicable***

We anticipate that Part I of the study will require between 60 and 90 minutes to consent and complete the questionnaires. Additionally, we predict that Part II of the study will require an estimated 55 minutes to complete

the pre-, mid-, and post- questionnaires total as well as 600 minutes to complete 10 treatment modules (estimated to take up to 60 minutes [including weekly homework] each to complete).

Questionnaires

MTurk Questions: (1) age; (2) relationship status; (3) are you an individual trying to get physically pregnant/ are you the partner of someone trying to get physically pregnant/I am not trying to get pregnant at this time; (4) how long have you been trying to get pregnant; (5) zipcode; (6) information on the Online Mind/body Fertility Program summary and would you be interested in participating in the online mind body treatment program. After participants complete the questions, they will be thanked for their participation and asked to email onlinemindbody@uvm.edu if they would like to participate in the rest of the study.

Screener questions include (1) do you and/or your partner meet criteria for an infertility diagnosis; (2) are you fluent in written/spoken English; and (3) do you have regular access to the Internet.

Demographic questions include age, gender, education level, employment status, yearly household income, relationship status and length of relationship, medical and medication related questions, infertility related questions, and general psychological functioning questions. Additionally, we will be capturing recruitment location by asking "how did you hear about us." Answer choices will be, you contacted me, search engine, social network, advertisement, friend, event, forum or blog, doctor, obgyn, fertility clinic. We will also keep track of zip code and specific name of answer choice (i.e., social network through facebook). This will allow us to better understand which recruitment efforts were more successful. Since this is a pilot study, the ability to recruit successfully and the ability to replicate successful recruitment efforts will be critical.

Health Risk Assessment (HRA): General health questions regarding risk and health history/behaviors. Moderate exercise was defined by (Center for Disease Control and Prevention, 2011).

Difficulties in Emotional Regulation Scale (DERS; Gratz & Roemer, 2004) is a 36-item self-report questionnaire measuring problems with emotional regulation based on responses indicating impairment in six dimensions. The higher the score, the greater the individual's difficulty with emotional regulation. The DERS has been shown to have high internal consistency ($\alpha = .93$) and adequate internal consistency per factor subscale ($\alpha > .80$) in a sample of 357 adults.

Dyadic Adjustment Scale (DAS, Spanier, 1976) A 32-item questionnaire showing good validity, able to discriminate between married and divorced couples, and found to have an overall reliability of .96, using Cronbach's co-efficient alpha (DAS; Spanier, 1976).

Perceived Stress Scale (PSS; Cohen, 1988; Cohen, Kamarck, & Mermelstein, 1983) is a 10 item questionnaire measuring the present level of self-rated stress in the last month. Items were designed to assess how unpredictable, uncontrollable, and overloaded respondents find their lives. The PSS has shown to be reliable and valid with good internal consistency $\alpha > .85$.

The Fertility Problem Inventory (FPI; Newton, Sherrard, & Glavac, 1999) is a 46-item questionnaire that measures domains considered important in understanding specifically perceived infertility related stress. The FPI has five subscales which include social concern, sexual concern, relationship concern, need for parenthood, and rejection of childfree lifestyle as well as a overall global index of infertility-related stress. Cronbach's alpha coefficients of internal consistency range from .77 to .93 for all subscales. Test-retest reliability coefficients for global stress were .83 for women and .84 for men.

Factors Affecting Fertility Scale (FAFS; Bunting, Boivin, 2008) is a 30-item questionnaire that measures domains of risk, myths, and health habits for fertility. Although research has begun on the development and testing of this scale, with some preliminary norms, more research is needed in regards to internal consistency and test-retest reliability.

The Brief Symptom Inventory (BSI; Derogatis, 1975; Derogatis & Melisaratos, 1983). The BSI is a 53-item self-report symptom scale encompassing 9 primary symptom dimensions. We are specifically interested in utilizing the items pertaining to general anxiety: nervousness or shakiness inside, suddenly scared for no reason, feeling fearful, feeling tense or keyed up, spells of terror or panic, feeling so restless you couldn't sit still. Internal consistency resulted in alpha ranges from .71-.85.

Fertility Quality of Life Questionnaire (FertiQol; Boivin, Takefman, Braverman, 2011) is a 36-item questionnaire that is divided into 4 domains (overall, personal, interpersonal and healthcare) and 9 dimensions (emotional, psychological, physical, values, partner relationship, social network, occupational/work, medical and psychoeducational. We will be using the domains specific to healthcare, specifically the "optional treatment qualify of life that includes 10 items on treatment environment and treatment tolerability. These domains have shown good reliability ranging from .72 (treatment tolerability) to .84 (treatment environment)

Part I--End of Survey Question: To assess if participants would be interested in treatment in general and/or Part II of the study, participants will be asked the following questions: (1) would you be interested in an in-person treatment protocol that could possibly reduce anxiety and depressive symptoms, and stress? Yes/No; (2) Would you be interested in an online treatment protocol that could possibly reduce anxiety and depressive symptoms, and stress? Yes/No; (3) If you answered No to either of these questions, could you provide the main reason you would not be interested: - it would not be helpful – I currently am not anxious, depressed, or stressed therefore do not think I need therapy – I do not have the time –other [please indicate your reason]; (4) If you qualify, would you be interested in engaging in Part II of this study that involves the potential of completing a online Mind/Body treatment program.

Five-Factor Mindfulness Questionnaire (FFMQ; Baer, Smith, Hopkins, Krietemeyer, & Toney, 2006). The FFMQ is a 39-item self-report measure used to assess a trait-like tendency to be mindful in daily life. It has five subscales including observe (i.e., notice or attend to internal and external stimuli), describe (i.e., mentally note stimuli with words), awareness (i.e., attend to their actions in the moment), non-judgmental (i.e., refrain from evaluating inner experiences), and non-reactive (i.e., not get caught up with their thoughts and feelings, allowing them to come and go). Examples of the five subscales include: "when I'm walking, I deliberately notice the sensations of my body moving" (observe), "I'm good at finding words to describe my feelings" (describe), "when I do things, my mind wanders off and I'm easily distracted" (awareness), "I criticize myself for having irrational or inappropriate emotions" (non-judgmental), and "I perceive my feelings and emotions without having to react to them" (non-reactive). Each question on the FFMQ is rated on a 5-point Likert scale ranging from 1 ("never or very rarely true") to 5 ("very often or always true"), with higher scores indicating greater mindfulness. All subscales have shown adequate to good internal consistency with alpha coefficient scores ranging from $\alpha = .75$ to .91. Test-retest reliability on the FFMQ has yet to be reported. The FFMQ has shown to have significant relationships in the predicted directions with a variety of related constructs (e.g., difficulties in emotion regulation, neuroticism, psychological symptoms; Baer et al., 2006). Furthermore, an increase in FFMQ scores in general, have found to fully mediate the relationship between total formal practice meditation time and perceived stress as well as psychological symptoms (Carmody & Baer, 2008).

MINI International Neuropsychiatric Interview for DSM-IV (MINI; Sheehan et al., 1998). The MINI is a standardized structured diagnostic interview that can evaluate for many psychiatric diagnoses meeting DSM-IV-TR criteria. The MINI assesses for a range of psychiatric conditions, include anxiety, mood, substance, eating, personality as well as suicidality and psychosis. Interrater reliability is good with all of the kappa values above 0.75, and with 70% of DSM-IV diagnoses as assessed by the MINI having a kappa value of .90 or higher. Test-retest kappa values, except for current mania (.45) are good with the majority of values being above .75. The MINI has on average shown adequate concordance with Structured Clinical Interview for DSM-III-R interviews and the Composite International Diagnostic Interview diagnoses.

Beck Anxiety Inventory (BAI; Beck et al., 1988; Beck, Steer, Beck, & Newman, 1993)**.** The BAI is a 21-item self-report measure used to assess somatic or panic-related anxiety symptoms. Example items include "feeling hot," "nervous," and "fear of losing control." Individuals are asked to rate their symptoms of anxiety over the past week on a 4-point Likert scale from 0 ("not at all") to 3 ("severely; I could barely stand it"). The BAI has excellent

internal consistency (α = .92) and good test-retest reliability after 1-week, r = .75 and convergent validity (Beck et al., 1988). Research has shown people who meet DSM criteria for an anxiety disorder have a mean score of 10.3 (*SD*= 7.5) or higher on the BAI (e.g., Leyfer, Ruberg, & Woodruff-Borden, 2006; Muntingh et al., 2011). In fact, one study determined that the cut-off score of 5.50 on the BAI for any anxiety disorder has a sensitivity of .76 and a specificity of .77, suggesting that a score greater than 5 on the BAI will identify 76% of those with a panic disorder and exclude 77% of those without (Leyfer et al., 2006). Therefore, a score of 8 or higher (8-15 = mild anxiety; Beck & Steer, 1999) is an appropriate cut-off that has shown to be consistent with a variety of anxiety disorders (e.g., Leyfer et al., 2006; Muntingh et al., 2011).

Beck Depression Inventory – 2nd Edition (BDI-II; Beck, Steer, Ball, & Ranieri, 1996)**.** The BDI-II is a 21-item self-report measure used to assess cognitive, affective, and physical depressive symptoms. Questions include content such as sadness, guilt, disappointment, and loss of energy. Participants are asked to rate each item on a 4-point Likert scale from 0 (not endorsed) to 3 (endorsed at maximum severity). The BDI-II has shown to have good validity (α = .90) and high one-week test-retest reliability, r = .93 (Beck et al., 1996).

Ongoing assessments. Participants will be asked at the beginning of every session if they completed assigned practice exercises at home since the last module. Additionally, at the end of each treatment module participants will be asked to comment on their ability to complete the module, including ease (0 "easy", 1 "moderately challenging", 2 "challenging") and helpfulness of module (0 "completely unhelpful", 1 "slightly helpful", 2 "moderately helpful", 3 "very helpful").

Additional Mid-Treatment Questions. Participants will be asked additional questions regarding (1) pregnancy status; (2) changes in psychotropic medications; (3) if they began other mental health treatments; (4) if they began infertility treatment.

Credibility/Expectancy for Improvement Scale (C/EIS; Borkovec & Nau, 1972; Devilly & Borkovec, 2000). The C/EIS is a five-item questionnaire designed to assess the credibility and the expectancy for treatment. The C/EIS has been revised to be appropriate for this sample, which includes items such as "how logical does this type of treatment seem to you" and "how confident would you be that this treatment would be successful in eliminating symptoms related to anxiety and/or depression." Questions are rated on a 10-point Likert scale. This scale has demonstrated factors that are stable across multiple populations, high internal consistency (α = .85) and good test-retest reliability (r = .83; Devilly & Borkovec, 2000).

The Client Satisfaction Inventory Short-Form (CSI-SF; McMurtry & Hudson, 2000). The CSI-SF is a 9-item self-report scale that assesses the satisfaction of the participant with the effects of the treatments. Participants are asked to rate the way they feel about the services they received on a 7-point Likert scale, ranging from 1 ("none of the time") to 7 ("all of the time"). Based on a scoring algorithm provided by McMurtry and Hudson (2000) the total scores range between 0 and 100, with higher scores indicating more satisfaction. This measurement has shown to have high internal consistency (α = .89).

Additional Post-Treatment Questions . Participants will be asked additional questions regarding (1) pregnancy status; (2) changes in psychotropic medications; (3) if they began other mental health treatments; (4) if they began infertility treatment. Participants will also be asked about helpfulness of treatment (0 "completely unhelpful", 1 "slightly helpful", 2 "moderately helpful", 3 "very helpful"), satisfaction with length of treatment (0 "too long", 1 = "too short", 2 = "right length of time") and two open-ended questions inquiring about advantages and disadvantages of internet-supported practice sessions (Cook & Doyle, 2002).

Part III Additional Questions: Participants will be asked questions related to infertility treatment/outcome and their chose to start/complete/discontinue treatment. These questions have been compiled from previous research on fertility treatment and discontinuation of fertility treatment (see **Part III Questionnaire**).

Measures to be Administered

	Time	Part I	Part II: Pre-	Part II: Treatment 7	Part II: Mid-Treatment	Part II: Post-Treatment	Part III

			Treatment	Weeks			
BAI	2 min	X	X	X	X	X	X
BDI	2 min		X		X	X	X
BSI	1 min	X				X	
C/EIS	1 min		X				
CSI-SF	1 min					X	
DAS	1 min	X				X	
Demographic Questions	15 min	X					
DERS	3 min	X			X	X	
FAFS	3 min	X				X	
~~FertiQol~~	~~1 min~~	~~X~~				~~X~~	~~X~~
FFMQ	3.5 min		X		X	X	
FPI	4 .5 min	X				X	X
~~HRA~~	~~1 min~~	~~X~~				~~X~~	
~~Mini~~	~~15 min~~	~~X~~				~~X~~	
Part I - Screener Questions	2 min	X					
Part I –End of Survey Questions	1 min	X					
Part II - Mid-Tx Assessment	1 min				X		
Part II – Treatment Satisfaction Questionnaire	3 min					X	
Part II -Ongoing Assessment	2 min			X	X		
Part III - Questions – DTS	1 min						X
PSS	1 min	X				X	

***Statistical Considerations:** Delineate the precise outcomes to be measured and analyzed. Describe how these results will be measured and statistically analyzed. Delineate methods used to estimate the required number of subjects. Describe power calculations if the study involves comparisons. Perform this analysis on each of the primary and secondary objectives, if possible.*

Sample size for Part I of the study was primarily determined based on the estimated number of participants needed for Part II of the study. Based on our previous feasibility study at Fletcher Allen we were able to recruit 177 women, where 138 completed the questionnaires, and of these 30% of women had heightened anxiety. Therefore it was originally estimated that 150 women (and their partners) recruited for Part I would result in 74 participants that meet criteria for Part II. Part II's sample size was determined based on feasibility and recommendations for pilot studies preceding clinical trials (Rounsaville, Carroll, & Onken, 2001). However, since recruitment procedures have drastically changed from the initial pilot study and recruitment has opened up to a much larger area, it is estimated we may need up to 300 women in order to result in the needed 74 participants for Part II.

Part I: We will assess the descriptive statistics (i.e., averages, percentages) of this population including those that score high on the previously stated measurements (see Measurement section above).

Part II: Randomization will be determined by using a random number generator via Random.org. This list will be pre-generated by a researcher other than the Principal Investigator, and this researcher will be responsible for indicating participant randomization group status. Participants will be evenly distributed across groups so that twenty participants will be in each condition. Participants will be informed during the consent process that they will have equal odds of being assigned to one of two groups (i.e., treatment or wait-list).The pilot data will be used

to estimate effect size for the impact of the mind-body intervention on BAI scores. First, enrollment and pre-treatment characteristics of the sample will be examined. Next, descriptive statistics will be provided on all outcome variables as well as other variables including expectancy, satisfaction, number of treatment modules completed, and meeting of exclusion criteria at some point throughout the study (i.e., became pregnant, started infertility treatment, changed psychotropic medication, and began additional psychotherapy). Effect size calculations will be conducted in order to assess improvements in anxiety, depression, emotion regulation, and mindfulness skills comparing the treatment group to the wait-list group. Clinical significance of treatment effects, Reliable Change Index, will be calculated in order to test that change attributed to treatment is most likely not due to chance.

Part III: We will also analyze the data with multiple logistic regressions to identify variables related to treatment outcomes (i.e., pregnancy, number of treatment cycles, and drop-out rates). Predictors for these analyses include psychological symptoms, difficulties in emotion regulation, stress, relational functioning, partners psychosocial functioning (when applicable), and treatment group.

Confidentiality Measures and Secure Storage of Data or Tissue: *Describe how the data/tissue will be collected. Will there be identifiers or will the data/tissue be coded? Describe where the data/tissue will be stored and how it will be secured. Describe who will have access to the data/tissue or the codes. If subject data/tissues with identifiers will be released, specify to whom. Describe what will happen to the data/tissues when the research has been completed.*

☐ ***Not Applicable***

Upon entering the study, participants will be assigned a random study ID. This ID will allow us to connect the online data with the individual for the purpose of (1) module/survey reminders, and (2) safety check-ins. The same identification key will be used for the online survey (see **Online/Email Instructions**). The researcher will electronically record the name and email address of the participant, partners name (when applicable), date they initially contacted us, and study ID. This electronic document will be password protected and only accessible by the research coordinator. This document will allow the assigned researchers to link the multiple surveys with specific participants as well as link participants with their partners that participate in the study.

MTurk: Participants will be recruited from and compensated via MTurk, but their responses will not be connected in any way to their MTurk accounts. This confidentiality, which will make it impossible to identify individual participants, will be accomplished by giving participants a link on MTurk to Limesurvey. After the completion of the brief screener, they will be given a completion code to enter into MTurk. That code is not associated or stored with the participant's Limesurvey screener and will not be stored or retained by Limesruvey in any way, and can thus not be used to associate MTurk account information with survey responses. It is thus impossible to identify any participant's responses.

The data provided online will be stored on a UVM LimeSurvey account and saved in an isolated password-protected folder on a backed-up and secure UVM network storage account. Once data from the questionnaires are entered in a SPSS data file, the token will allow us to connect the data for that participant. The SPSS files are stored on a secured UVM networked drive that can only be accessed by the research staff. The SPSS file will not contain identifying information.

Risks/Benefits: *Describe any potential or known risks. This includes physical, psychological, social, legal or other risks. Estimate the probability that given risk may occur, its severity and potential reversibility. If the study involves a placebo or washout period, the risks related to these must be addressed in both the protocol and consent. Describe the planned procedures for protecting against or minimizing potential risks and assess their likely effectiveness. Where appropriate, discuss plans for ensuring necessary medical or professional intervention in the event of adverse effects to the subjects. Discuss the potential benefits of the research to the subjects and others. Discuss why the risks to the subjects are reasonable in relation to the anticipated benefits to subjects and others. Discuss the importance of the knowledge gained or to be gained as a result of the proposed research and why the risks are reasonable in relation to the knowledge that reasonably may result. If there are no benefits state so.*

Risks

Some of the questions are personal in nature and may produce some social discomfort in subjects. Since the participants are engaging in an infertility assessment it is anticipated that they already expect to have a discussion involving certain aspects of their psychosocial and sexual functioning. Also, participants will be told that they will

be asked to complete self-reported questionnaires about psychosocial functioning during the informed consent procedures. Utilizing online self-reported questionnaires allows for greater sense of privacy and reduces potential embarrassment and discomfort (as compared to face-to-face interviews). Also, by informing participants about the nature of the study we increase the likelihood that only people that feel comfortable answering these questions will participate. To further reduce any potential embarrassment or discomfort, participants will be instructed in the beginning of the study that they may choose not to answer any question or stop at any time, should they feel uncomfortable, without consequence to the study, their relationship with the experimenter, and the research team.

Participants will be informed that all study data will be kept confidential. Additionally, we will be collecting minimal PHI and we obtained a NIH Certificate of Confidentiality to minimize the possibility of a breach.

We will do our best to protect the information we collect from the participant and their medical record. Information that identifies the participant will be kept secure and restricted. All of the participants' information will be kept in electronic format that will be password protected. However, there is a potential risk for an accidental breach of confidentiality. In the case that an accidental breach does occur, we will notify the Informational Review Board who will help us identify the most appropriate and ethical approach to respond to the problem.

As with any psychotherapy, participants may experience discomfort or embarrassment answering some of the questions involved. Furthermore, participation in psychotherapy may bring up issues that the participant was unaware of, such as distressing thoughts and/or feelings, or they may decide to make important life changes. However, these risks are no different than any other psychotherapy offered in the community and participants can decline to participate at any time. Specific to the Mind/Body Treatment protocol, an introduction to Hatha Yoga and instructions to engage in light stretching is built into the protocol. As with any exercise, there is a slight possibility of injury. Participants will be encouraged during the consent process to consult their physician and follow their physicians' advice prior to engaging in any exercises. Additionally, this protocol includes health related information including nutrition and other lifestyle behaviors related to fertility. Again, participants will be encouraged to contact their physician before making any lifestyle related changes.

A risk that may be specific to online psychotherapy, is that intervening when someone is a danger to themselves or others will not be conducted immediately when someone is completing the module. Participants will have been provided with psychological referrals including referrals for emergency numbers that assist in crisis (i.e., suicidal ideation/intent, etc). Additionally, we will be screening out any participants that meet criteria for "high risk" for suicidality based on the MINI Psychiatric Interview (see Measurements). Any participants that endorse suicidal ideation/intent or other crisis information in the modules/through email that is not specifically assessed for will be encouraged through email to contact National Crisis Line (http://www. mpreventionlifeline.org/; 1-800-273-8255) and/or Crisis Call Center (http://www.crisiscallcenter.org/contactus.html; 1-800-273-8255).

<u>Guidelines for Addressing Suicidal Ideation</u>

Part I: As described in the protocol the participants complete the Beck Depression Inventory and MINI Psychiatric Interview online that both ask questions about self-harm. Since this protocol is entirely online, can be completed any time day/night, and for the purpose of privacy we are attempting to limit the amount of PHI we collect on each participant we believe that the most ethical practice would be to encourage participants that do endorse suicidal ideation/intent to contact the National Crisis Line (http://www.suicidepreventionlifeline.org/; 1-800-273-8255). The great thing about using Limesurvey is that we can build this into our logic model and automatically deliver this message if someone were to indicate suicidal ideation/intent. If at any other time in the protocol someone indicates suicidal ideation or some other emotional crisis/issue (i.e., homicidal ideation) we will email the individual as soon as we become aware of this information and encourage them to contact either the (http://www.suicidepreventionlifeline.org/; 1-800-273-8255) or the Crisis Call Center (http://www.crisiscallcenter.org/contactus.html; 1-800-273-8255), which deals with more general crisis. Although we do not foresee participants endorsing/sharing information regarding abuse or intentions/thoughts of harming others, it is always a possibility in any study that is working with people. Since we are collecting minimal PHI as a way to protect participants data, we do not believe that we have enough information to report. We plan to email all individuals that participate in the study psychological resources (see revised document: Psychological Referrals),

including individuals that do not qualify for our study because they are a "high" risk for suicidality as assessed by the MINI (see Questionnaire section in Protocol). From our experience recruiting in previous studies, we have not successfully been able to establish contact with many of our participants in order to further assess them. Providing participants with 24/7 emergency contact resources will be the fastest and most comprehensive way to address this.

Benefits

Part I: There are no direct benefits to the participants due to participation in this study. The knowledge to be gained from this study may be used to better understand the role of psychological, emotional, and relational functioning of both couples seeking out medical treatment for infertility. In addition, this study may begin to identify those factors present at first contact and/or throughout treatment with couples that would be most indicated for psychological referral.

Part II: No controlled studies exist that support the efficacy of this online Mind-Body treatment program for infertile couples struggling with anxiety and/or depression. However, research does exist that show that this protocol delivered in-person in a group setting has been effective in reducing anxiety and depressive symptoms. Participants will receive 10 online modules that closely matches the content of the in-person treatment manual (only modification is that which is needed to make it relevant to an online modality). We hope that the participants will learn new skills and behaviors that can help reduce anxiety and/or depression

Therapeutic Alternatives: *List the therapeutic alternatives that are reasonably available that may be of benefit to the potential subject and include in the consent form as well.*

☐ ***Not Applicable***

The techniques we are utilizing in our treatment belong to the tradition of mindfulness-based and cognitive-behavioral therapies. Although "online therapies" currently exist for infertilitycurrently there are no evidence-based online therapies available to the public to address anxiety and/or depressive symptoms in infertile couples. Currently the closest Mind-Body Program available for infertile couples is located outside of Boston, Massachusetts. Participants not interested in participating in the study will be provided with information on how to locate a therapist in there area and the location for Mind-Body in-person group treatment program in Massachusetts at the Domar Center (see **Psychological Referral List**).

Data Safety and Monitoring: *The specific design of a Data and Safety Monitoring Plan (DSMP) for a protocol may vary extensively depending on the potential risks, size, and complexity of the research study. For a minimal risk study, a DSMP could be as simple as a description of the Principal Investigator's plan for monitoring the data and performance of safety reviews or it could be as complex as the initiation of an external, independent Data Safety and Monitoring Board (DSMB). The UVM/FAHC process for review of adverse events should be included in the DSMP.*

To ensure accurate data collection, all research assistants will be instructed to check all questionnaires to ensure they are completed. For the purpose of maintaining data integrity, for surveys that are incomplete (i.e., missing answers) an electronic survey will be sent to the participant that includes the specific missing measurement(s). In order to better determine whether the questionnaires were intentionally skipped versus incomplete, each item will specifically have the option "skip." Participants will be emailed and informed that they either missed some items mistakenly or purposively and we are requesting that if they missed the questions by mistake that they complete the questionnaire now (see **Online Information/Email**).

Research data that is obtained through the online surveys will be stored on a UVM LimeSurvey account and saved in an isolated password-protected folder on a backed-up and secure UVM network storage account. Only the Principal Investigator and the key personnel will have access to this password. Also, data on the secure networked drive and saved in the password-protected account will not have identifying information outside the demographics questions. Data is saved on a daily basis. From the password-protected folder, data will be transferred into SPSS files that will be housed on a server that can be accessed only by the Sexual Health Research Clinic staff. LimeSurvey is hosted by UVM and was selected to replace the previously-used Perseus system as the preferred survey collection tool available for use by UVM faculty. A password protected electronic document will be created to store participants names, email addresses and study ID. Only the study coordinator will have access to this document.

MTurk: Participants will be recruited from and compensated via MTurk, but their responses will not be connected in any way to their MTurk accounts. This confidentiality, which will make it impossible to identify individual participants, will be accomplished by giving participants a link on MTurk to Limesurvey. After the completion of the brief screener, they will be given a completion code to enter into MTurk. That code is not associated or stored with the participant's Limesurvey screener and will not be stored or retained by Limesruvey in any way, and can thus not be used to associate MTurk account information with survey responses. It is thus impossible to identify any participant's responses.

A data quality team comprised of the study coordinators is responsible for random checks of data quality computerized copy of data. The SPSS file containing all data lives in a password protected UVM approved and secure server backed up every night. In addition to the data safety team, the PI and Co-Investigator will conduct random quality checks to ensure the quality of the data entered.

The data file with the raw data will be kept separately from data analyses and no new data will be entered or saved in this file to ensure that the data remains pristine. A new file will be created with the total scores and factor scores derived from the raw data and this file will be used for data analyses. No raw data will be modified in this file.

Adverse Event and Unanticipated Problem (UAP) Reporting: *Describe how events and UAPs will be evaluated and reported to the IRB. All protocols should specify that, in the absence of more stringent reporting requirements, the guidelines established in the Committees on Human Research "Adverse Event and Unanticipated Problems Reporting Policy" will be followed. The UVM/FAHC process for review of adverse events and UAPs to subjects or others should be included in the DSMP.*

Any deviation for the protocol or concerns reported by the participants will be reviewed first by the PI (Alessandra Rellini, PhD) then reported to the IRB in case the event is a sign of potential distress in the participant. All events that are considerable variations from the protocol or describe events that are unexpected will be summarized in the IRB review even if they did not result in any negative discomfort for the participant. In case the person expresses distress associated with their participation in the study, the PI will do all of the following: (1) assess the cause of distress (2) provide a referral for a counselor to discuss their difficulties, (3) identify a plan to prevent similar outcomes to future participants, and (4) report the incident and the plan to the IRB.

Unanticipated problems will be recorded and Dr. Rellini or the study coordinators will collect exhaustive information on the causes and effects of the problem. The incident will be discussed among the research team within 24 hrs from the report and a plan will be determined. If the problem is considered significant the IRB will be notified within 24 hrs and a specific plan of action will be reviewed to (1) protect the person(s) affected, and (2) prevent this from happening in the future. If the problem is not significant it will be filed in a document that is created to monitor any unexpected problems that need to be considered before the development of future research proposals.

The serious adverse events form will be used by the study staff to report any adverse events caused by the treatment intervention. There is minimal risk of adverse events expected based on data from the in-person treatment Mind-Body treatment trials previously conducted (Domar, Clapp, Slawsby, Dusek, et al., 2000; Domar et al., 2011).

Withdrawal Procedures: *Define the precise criteria for withdrawing subjects from the study. Include a description of study requirements for when a subject withdraws him or herself from the study (if applicable).*

Participants can withdraw their consent at any time. Participants will be informed that if they no longer wish to complete the study they can withdraw at any point. If participants decide to terminate the study, they will be informed that this can occur without consequences to their care outside this study and their relations with the faculty, staff, and administration the University of Vermont. Participants will be informed that they can also choose to not answer particular question but not withdraw completely from the study and that this can occur without consequences.

If they choose to withdrawal their consent they must do so by emailing the Principal Investigator. If the participants withdraws their consent, the information the participant provided up to that point may still be utilized

for analyses. The participants' data will be retained for a period of five years after submission of the final report for the project for which the data were collected according to the UVM Policy on Retention of Data. If they withdraw, they may no longer participate in the research study.

Should new scientific developments occur that indicate the treatment is not in participants' best interest, or should the therapy leader feel that this treatment is no longer in a participant's best interest, the treatment will be stopped. In addition, the researcher may discontinue any participant's participation in this study at any time.

Sources of Materials: *Identify sources of research material obtained from individually identifiable human subjects in the form of specimens, records or data. Indicate whether the material or data will be obtained specifically for research purposes or whether use will be made of existing specimens, records or data.*

Data will be provided by the participants only.

DRUG AND DEVICE INFORMATION

Investigators are encouraged to consult the FAHC Investigational Pharmacy Drug Service (847-4863) prior to finalizing study drug/substance procedures.

Drug (s) [X] ***Not applicable***

Drug name – generic followed by brand name and common abbreviations. Availability – Source and pharmacology; vial or product sizes and supplier. If a placebo will be used, identify its contents and source. (attach investigational drug brochure)

Preparation: Reconstitution instructions; preparation of a sterile product, compounded dosage form; mixing guidelines, including fluid and volume required. Identify who will prepare.

Storage and stability – for both intact and mixed products.

Administration – Describe acceptable routes and methods of administration and any associated risks of administration.

Toxicity – Accurate but concise listings of major toxicities. Rare toxicities, which may be severe, should be included by indicated incidence. Also adverse interactions with other drugs used in the protocol regimen as well as specific foods should be noted. Address significant drug or drug/food interactions in the consent form as well. List all with above details.

Is it FDA approved: (include FDA IND Number)

1. *in the dosage form specified? If no, provide justification for proposed use and source of the study drug in that form.*
2. *for the route of administration specified? If no, provide justification for route and describe the method to accomplish.*
3. *for the intended action?*

Device (s) [X] ***Not applicable***

Device name and indications (attach investigational device brochure)

Is it FDA approved: (include FDA IDE Number)

1. *for indication specified? If no, provide justification for proposed use and source of the device.*

Risk assessment (non-significant/significant risk) - PI or sponsor needs to assess risk of a device based upon the use of the device with human subjects in a research environment.

SUBJECT CHARACTERISTICS, IDENTIFICATION AND RECRUITMENT

Subject Selection: *Provide rationale for subject selection in terms of the scientific objectives and proposed study design.*

Participants are to be recruited from physical and online locations specifically to target infertile couples. The general population are excluded (i.e., not infertile), because the research questions specifically entails individuals with an infertility diagnosis.

Vulnerable Populations: *Explain the rationale for involvement of special classes of subjects, if any. Discuss what procedures or practices will be used in the protocol to minimize their susceptibility to undue influences and unnecessary risk (physical, psychological, etc.).*

X ***Not applicable***

Number of Subjects: *What is the anticipated number of subjects to be enrolled at UVM/FAHC and in the case of a multi-center study, with UVM/FAHC as the lead, the total number of subjects for the entire study.*

MTurk: A total of 350 women will be recruited from MTurk to complete the brief screener.

Part I & III: Up to 300 women and their partners (when applicable) will be recruited for the first part of this study.

Part II: A total of 74 women and their partners will be randomized for Part II of the study. Half of the participants will be randomized to the treatment group whereas half will be randomized to the wait-list group.

Inclusion/Exclusion Criteria: *Eligibility and ineligibility criteria should be specific. Describe how eligibility will be determined and by whom. Changes to the eligibility criteria at a later phase of the research have the potential to invalidate the research.*

MTurk: Inclusion Criteria
- Women only
- Infertility diagnosis
- Trying to get pregnant
- 18 years or older
- Read and write English
- Only opening it up to women from the United States

Online Eligibility Screener
- Women and Men
- Infertility diagnosis
- Trying to get pregnant
- 18 years or older
- Read and write English

Part I: Inclusion Criteria
- Infertility diagnosis
- Trying to get pregnant
- Women (18 years or older) and their partners
- Access to the internet
- Read and write English

Part II: Inclusion Criteria
Eligibility for Part II will be determined based on the data given for Part I. Only participants that indicate that they would be interested in a Mind-Body treatment and meet study criteria will be provided with a link to the Part II online consent process.
- Meet criteria for Part I
- Primary infertility diagnosis (i.e., never given birth)
- Woman or partner of woman who qualifies for Part II
- No current diagnosis for an active psychotic disorder, eating disorder, substance abuse or dependence
- Not reporting current suicidal ideation/intent (assessed by the MINI as "high" risk)
- No psychotropic medication changes in the last four weeks

- Has not completed a formal mind/body program focused on infertility that included relaxation, yoga, mindfulness, cognitive restructuring, stress reduction strategies, listening and communication skills, goal setting and assertiveness training.
-

Part III: Inclusion Criteria

- All participants that complete Part I will be invited to complete Part III.

Inclusion of Minorities and Women: *Describe efforts to include minorities and women. If either minorities or women are excluded, include a justification for the exclusion.*

Recruitment efforts will be focused on the individuals that attend the fertility clinic, which from our experience recruiting in the past are primarily women. However it is hoped that the partners will also attend and therefore will recruit as many men as possible. Recruitment efforts will be equal across ethnicity. It is anticipated that the sample will roughly approximate the patient population seen in New England.

Inclusion of Children: *Describe efforts to include children. Inclusion is required unless a clear and compelling rationale shows that inclusion is inappropriate with respect to the health of the subjects or that inclusion is inappropriate for the purpose of the study. If children are included, the description of the plan should include a rationale for selecting or excluding a specific age range of children. When included, the plan must also describe the expertise of the investigative team in working with children, the appropriateness of the available facilities to accommodate children, and the inclusion of a sufficient number of children to contribute to a meaningful analysis relative to the purpose of the study.* ***If children are excluded*** *then provide appropriate justification. Provide target accrual for this population.*

Children are excluded because they will not qualify for the inclusion criteria of meeting criteria for an infertility diagnosis and wanting to get pregnant.

For protocols including the use of an investigational drug, indicate whether women of childbearing potential have been included and, if not, include appropriate justification.

If HIV testing is included specifically for research purposes explain how the test results will be protected against unauthorized disclosure. Include if the subjects are to be informed of the test results. If yes, include the process and provision for counseling. If no, a rationale for not informing the subjects should be included.

X	***Not applicable***

Recruitment: *Describe plans for identifying and recruitment of subjects. All recruitment materials (flyers, ads, letters, etc) need to be IRB approved prior to use.*

Part I: We will be inviting fertility clinics in Vermont, Connecticut, Massachusetts, Rhode Island, New Hampshire, and parts of New York to help us in recruiting for this study by contacting the directors/managers of each of the clinics and informing them about the study and how they can get involved (see Letter to Clinic/Information Sheet). Specifically, we will be providing clinics with information about the study, along with recruitment flyers to post in their clinic and include in new patient packets (see Recruitment Flyers) and text to post on their website (when applicable; see text in Recruitment Flyers). Additionally, staff will be asked to inform prospective subjects about the availability of the research and provide prospective subjects with information about the research by providing them with a study flyer with the relevant recruitment information within the new patient packet for infertility patients (when applicable). All screening will be conducted online. Individuals will be directed to email the researchers and interested individuals will be given a token specific link that will direct them to the Limesurvey questionnaires.

Part II: Participants that meet study eligibility will be provided with additional information regarding the second part of the study online. A total of forty participants will be invited to participate in Part II of the study. Participants that agree to participate will be able to give their consent by clicking yes/no through an online survey (using LimeSurvey). After participants agree to Part II of the study they will be randomized to either the treatment or wait-list group.

Part III: All participants that complete Part I will be eligible for Part III.

FINANCIAL CONSIDERATIONS

Expense to Subject: *If the investigation involves the possibility of added expense to the subject (longer hospitalization, extra studies, etc.) indicate in detail how this will be handled. In cases where the FDA has authorized the drug or device company to charge the patient for the experimental drug or device,* ***a copy of the authorization letter from the FDA or sponsor must accompany the application. Final approval will not be granted until the IRB receives this documentation.***
There are very limited circumstances under which study participants may be responsible (either directly or via their insurance) for covering some study-related expenses. If the study participant or their insurer(s) will be billed for any portion of the research study, provide a justification as to why this is appropriate and acceptable. For example, if the study involves treatment that is documented standard of care and not investigational, state so. In these cases, the protocol and the consent should clearly define what is standard of care and what is research.

No expense for participation is anticipated other than the participants' own time.

Payment for participation: *Describe all plans to pay subjects, either in cash, a gift or gift certificate. Please note that all payments must be prorated throughout the life of the study. The IRB will not approve a study where there is only a lump sum payment at the end of the study because this can be considered coercive. The amount of payment must be justified. Clarify if subjects will be reimbursed for travel or other expenses.*

[ ] ***Not applicable***

MTurk: For participants that complete the MTurk Screener they will receive 20 cents in their MTurk account.

Part I: .For participants that complete Part I we will enter them into a drawing to get a $25 e-gift certificate to Amazon.com. A drawing will occur each time a total of 25 people complete the study and continue until we complete recruitment.

Part II: Control participants will receive $10 Amazon gift certificate for completing the initial pre-treatment questionnaire and $15 Amazon gift certificate upon completion of the online post-treatment questionnaires as an incentive to complete the study. Similar compensation has been noted in other RCT's with the mind-body intervention (Domar et al., 2011). Since the mind-body intervention currently is offered as an in-person group format commercially for $780 ("FAQ's," 2013), participants randomized to the treatment group will receive the treatment itself as compensation.

Collaborating Sites*. When research involving human subjects will take place at collaborating sites or other performance sites when UVM/FAHC is the lead site, the principal investigator must provide in this section a list of the collaborating sites and their Federalwide Assurance numbers when applicable. (agreements may be necessary)*

[X] ***Not applicable***

INFORMED CONSENT

Consent Procedures*: Describe the consent procedures to be followed, including the circumstances under which consent will be obtained, who will seek it, and the methods of documenting consent.*

<u>Note</u>: Only those individuals authorized to solicit consent may sign the consent form confirming that the prospective subject was provided the necessary information and that any questions asked were answered.

Part I: Information about the study will be provided entirely online and that consent will be obtained online by asking participants to provide their consent for the study by clicking "yes" in the Limesurvey application (see Request for Waiver of Informed Consent/Authorization/Documentation). Participants will be encouraged to ask questions or raise concerns prior to consenting and will be given the option to click "I have questions/concerns" and provided with a comment box. A researcher will contact the individual by email to address the questions/concerns. Participants that are still interested after their questions/concerns are addressed will be redirected to the token-

specific link and requested to select “yes” indicating their consent.

Part II: Participants that meet eligibility criteria will be provided with the pertinent information regarding Part II of the study again, including information on the treatment, randomization, and online assessment schedule. Participants that agree to participate in the second part of the study will be asked to indicate their consent by checking “yes” or “no” through Limesurvey.

Part III: Participants that complete Part I of the study will be provided with the pertinent information regarding Part III of the study and asked to consent by checking “yes” or “no” through Limesurvey.

Information Withheld From Subjects: *Will any information about the research purpose and design be withheld from potential or participating subjects? If so, explain and justify the non-disclosure and describe plans for post-study debriefing.*

X	***Not applicable***

Consent, Assent, and HIPAA Authorization. *Specify the form(s) that will be used e.g. consent (if multiple forms explain and place identifier on each form), assent form and/or HIPAA authorization (if PHI is included). These form(s) must accompany the protocol as an appendix or attachment.*

See **Informed Consent** form and **Waiver of Informed Consent/Authorization/Documentation** form.

Attach full grant application, including budget information and/or any contract or draft contract associated with this application.
