# Supplementary material for: An internet-based mind/body intervention to mitigate distress in women experiencing infertility: A randomized pilot trial
Source: PLoS One. 2020 Mar 18;15(3):e0229379. doi: 10.1371/journal.pone.0229379 (PMC7080396; doi:10.1371/journal.pone.0229379)
Supplement: S1 File — (DOCX) [file pone.0229379.s003.docx]

**S1 File. For code, data, and presentations go to:** [**https://doi.org/10.17605/OSF.IO/FC2EJ**](https://doi.org/10.17605/OSF.IO/FC2EJ)**.**
